# Prevalence, awareness, treatment, and control rates of hypertension in patients hospitalized with atrial fibrillation in China: Findings from the CCC-AF project

**DOI:** 10.3389/fcvm.2022.970787

**Published:** 2022-08-01

**Authors:** Zhaoqing Sun, Yongchen Hao, Jun Liu, Na Yang, Yue Qi, Danqing Hu, Yiqian Yang, Haimei Wang, Dong Zhao, Caihua Sang, Jing Liu

**Affiliations:** ^1^Center for Clinical and Epidemiologic Research, Beijing Anzhen Hospital, Capital Medical University, Beijing Institute of Heart, Lung and Blood Vessel Diseases, Beijing Municipal Key Laboratory of Clinical Epidemiology, Beijing, China; ^2^Department of Cardiology, National Clinical Research Center for Cardiovascular Diseases, Beijing Anzhen Hospital, Capital Medical University, Beijing, China

**Keywords:** atrial fibrillation, hypertension, prevalence, awareness, treatment, control

## Abstract

**Background:**

The status of hypertension in patients with atrial fibrillation (AF) remains unknown in China.

**Methods:**

This study used data from patients hospitalized with AF recruited by the Improving Care for Cardiovascular Disease in China-AF (CCC-AF) project from 236 hospitals enrolled by geographic-economic level in China from 2015 to 2019. The prevalence, awareness, treatment, and control rates of hypertension in patients hospitalized with AF were estimated. Multivariable logistic regression was used to analyze the factors associated with uncontrolled hypertension.

**Results:**

Among 60,390 patients hospitalized with AF, the prevalence of hypertension according to the 2018 Chinese hypertension guidelines was 66.1%. The awareness, treatment, and control rates of hypertension were 80.3, 55.8, and 39.9%, respectively. Among patients treated for hypertension, the treatment control rate was 46.2%. These rates varied according to patient clinical characteristics and geographic regions. The young (18–44 and 45–54 years old), rural insurance, alcohol drinking, history of heart failure, valvular AF, first diagnosed AF, and permanent AF, were associated with uncontrolled hypertension. Under the 2017 American College of Cardiology/American Heart Association (ACC/AHA) hypertension guidelines, the prevalence of hypertension was 79.3%, and the control and treatment control rates dropped to 16.7 and 21.2%, respectively.

**Conclusion:**

Hypertension is common in patients hospitalized with AF in China. Although most patients were aware of their hypertensive status, the treatment and control rates of hypertension were still low. The management of hypertension in patients with AF needs to be further improved.

## Introduction

Atrial fibrillation (AF) has received increasing attention from the public due to its close association with adverse outcomes, such as stroke, heart failure and death (1, 2). In China, the estimated number of adults with AF increased from 4 million in 2008 to 7.9 million in 2014–2016 (3, 4). Hypertension has long been recognized as a risk factor for and a common comorbidity with AF, and it was found in 42.0–90.5% of patients with AF in prior studies (5–13). Poor control of hypertension may aggravate the adverse outcomes of AF through a variety of mechanisms, including left ventricular hypertrophy, kidney dysfunction, and cardiovascular and cerebrovascular disorders (14). Therefore, management of blood pressure (BP) has been recommended by current guidelines and consensus for treatment of patients with AF and hypertension, as it reduces the risk of embolism and bleeding (15–17). However, only 45.9–78.0% of patients with AF have well-controlled BP according to previous international multicenter registry studies (5–9). The control of hypertension is particularly important for patients with AF in China, as hypertension is the leading risk factor for all-cause mortality (18). Hypertension has been found in 55.5–72.5% of Chinese patients with AF according to local or regional registries (10–13). However, studies have not focused on the prevalence of hypertension in a large representative sample, and the awareness, treatment, and control rates of hypertension in patients with AF remain unknown. Given the current epidemic of AF and hypertension in China, obtaining contemporary data on the prevalence and control of hypertension in patients with AF is crucial for decision-making in clinical practice.

Therefore, we used data from the Improving Care for Cardiovascular Disease in China-Atrial Fibrillation (CCC-AF) project, a large national registration study, to obtain the latest estimates of the prevalence, awareness, treatment, and control rates of hypertension in patients hospitalized with AF in China.

## Materials and methods

### Study design and population

The CCC-AF project was jointly initiated by the Chinese Society of Cardiology (CSC) and the American Heart Association (AHA) in February 2015 and conducted by the Beijing Institute of Heart, Lung and Blood Vessel Diseases. Details on the design and methodology of the CCC-AF project have been published previously (19). Registration information can be found at the following URL: https://clinicaltrials.gov (Unique Identifier: NCT02309398). Hospital sampling frame was stratified by geographic-economic level to ensure national representativeness, with 10 and 1.7% of tertiary and secondary hospitals, respectively recruited in each stratum (Supplementary Table 1). A total of 236 hospitals (151 tertiary hospitals and 85 secondary hospitals) from seven regions of China (Northeast, Northwest, North, East, Central, South, and Southwest China) were included in the CCC-AF project, which collected data from February 2015 to December 2019. In this project, 10–20 patients (≥18 years of age) from each tertiary hospital and 5–10 patients from each secondary hospital whose discharge diagnosis included AF were enrolled in a consecutive manner each month. Patients with AF due to reversible conditions, such as untreated thyroid disease and pulmonary embolism, were excluded. The CCC-AF project was approved by the Institutional Review Board of Beijing Anzhen Hospital, with a waiver for informed consent (No. 2014018). A total of 61,136 patients hospitalized with AF were recruited during the study period, and 60,390 patients were included in this analysis, after excluding patients whose BP records at admission were missing (*n* = 746).

### Data collection

Clinical data, including demographic information, medical history, pre-hospital treatments, physical examinations at admission, the etiological diagnosis of AF, types of AF, and the principal discharge diagnosis were extracted from the patients' medical records (19). The collection of the abovementioned clinical data from patients with AF who met the inclusion criteria was completed by well-trained data entry personnel in each participating hospital using a standard web-based data collection platform (Oracle Clinical Remote Data Capture; Oracle Corporation, Redwood City, CA).

### Definitions

Hypertension was defined as systolic BP (SBP) ≥ 140 mm Hg or diastolic BP (DBP) ≥ 90 mm Hg measured at admission in patients hospitalized with AF or a documented history of hypertension, according to the Chinese Guidelines for the Management of Hypertension (20). The definitions of the prevalence, awareness rate, treatment rate, control rate, and treatment control rate of hypertension in patients hospitalized with AF in China have been summarized in Table 1. Meanwhile, the prevalence, control rate, and treatment control rate of hypertension were also estimated with cutoff values of SBP = 130 mm Hg and DBP = 80 mm Hg, according to the 2017 American College of Cardiology/American Heart Association (ACC/AHA) High Blood Pressure Guidelines (21). Prehospital antihypertensive drugs included angiotensin converting enzyme inhibitors (AECIs), angiotensin receptor blockers (ARBs), β blockers, calcium channel blockers (CCBs), diuretics, aldosterone receptor antagonists (ARAs), α blockers, and direct-acting vasodilators.

**Table 1:** Definitions of prevalence, awareness rate, treatment rate, control rate, and treatment control rate of hypertension in patients hospitalized with atrial fibrillation.

**Variables**	**2018 Chinese hypertension guidelines**	**2017 ACC/AHA hypertension guidelines**
Hypertension	SBP ≥ 140 mm Hg or DBP ≥ 90 mm Hg measured at admission, or a documented history of hypertension	SBP ≥ 130 mm Hg or DBP ≥80 mm Hg measured at admission, or a documented history of hypertension
Prevalence	The percentage of patients with hypertension out of the total number of patients hospitalized with AF	The percentage of patients with hypertension out of the total number of patients hospitalized with AF
Awareness rate	The proportion of patients who had previously known hypertension among all hypertensive patients hospitalized with AF	–
Treatment rate	The proportion of patients taking antihypertensive drugs prior to the current admission among all hypertensive patients hospitalized with AF	–
Control rate	The proportion of patients with SBP < 140 mm Hg and DBP < 90 mm Hg among all hypertensive patients hospitalized with AF	The proportion of patients with SBP < 130 mm Hg and DBP < 80 mm Hg among all hypertensive patients hospitalized with AF
Treatment control rate	The proportion of patients with SBP < 140 mm Hg and DBP < 90 mm Hg among hypertensive patients hospitalized with AF and on treatment with antihypertensive drugs	The proportion of patients with SBP < 130 mmHg and DBP < 80 mmHg among hypertensive patients hospitalized with AF and on treatment with antihypertensive drugs

ACC/AHA, American College of Cardiology/American Heart Association; SBP, systolic blood pressure; DBP, diastolic blood pressure; AF, atrial fibrillation.

Body mass index (BMI) was calculated as patient weight divided by the square of height (kg/m^2^). Underweight and obese individuals were defined as having BMIs of <18.5 kg/m^2^ and ≥28.0 kg/m^2^, respectively, according to the Chinese Guidelines on the Control of Overweight and Obesity (22). Definitions for other study variables are provided in Supplementary Table 2.

### Statistical analysis

Continuous variables with a normal distribution are expressed as the mean ± standard deviation and were compared using unpaired *t*-tests. Continuous variables with skewed distributions are expressed as medians and interquartile ranges and were compared using the Mann–Whitney *U*-test. Categorical variables are expressed as frequencies and percentages and were compared using the chi-square test or linear-by-linear association. Factors associated with uncontrolled hypertension in patients hospitalized with AF were assessed using two-level logistic regression (patient-level and region-level), and the multivariable model further adjusted for the following candidate variables: age group, sex, BMI category, medical insurance, smoking, alcohol drinking, coronary heart disease (CHD), heart failure, cerebrovascular disease, diabetes mellitus, previous bleeding, prehospital use of antihypertensive drugs, etiological diagnosis of AF, and types of AF. The variable assignments in the multivariable analysis are listed in Supplementary Table 3. BP levels for patients hospitalized with AF were divided into the following four groups: <130/ <80, 130–139/80–89, 140–159/90–99, and ≥160/100 mm Hg, regardless of antihypertensive treatments. For variables with missing data, we imputed the missing values of clinical characteristics using the sequential regression multiple imputation method in IVE ware software version 0.2 (Survey Research Center, University of Michigan, Ann Arbor, MI), except for BMI, for which 44.5% of values were missing. A sensitivity analysis was performed after excluding patients with missing BMI data. Both the missing rates of the study variables and the management of missing data are shown in Supplementary Table 4. All *P*-values were two-tailed, and *P* < 0.05 was considered statistically significant. Statistical analysis was performed with SPSS, version 25.0 (IBM Corporation, Armonk, NY, USA) and R version 4.1.3 (2020-03-10).

## Results

### Characteristics of study participants

Among the 60,390 patients hospitalized with AF, the median age was 70.0 (range: 62.0–78.0), and 54.3% were men. The mean values of SBP and DBP were 130.7 ± 21.0 mm Hg and 79.3 ± 13.9 mm Hg, respectively. Significant differences were observed between hypertensive and non-hypertensive patients hospitalized with AF in all characteristics except for the use of antiarrhythmic drug and electrical cardioversion, based on the Chinese diagnostic criteria. Similar results were found with the ACC/AHA diagnostic criteria, except for alcohol drinking and previous bleeding (Table 2). The proportions of patients hospitalized with AF with <130/ <80, 130–139/80–89, 140–159/90–99, and ≥160/100 mm Hg were 33.9, 26.3, 24.8, and 15.0%, respectively (Supplementary Figure 1).

**Table 2:** Clinical characteristics of patients hospitalized with atrial fibrillation by different diagnostic criteria.

**Characteristics**	**Total (*N* = 60,390)**	**2018 Chinese hypertension guidelines**			**2017 ACC/AHA hypertension guidelines**		
		**With hypertension** **(*n* = 39,917)**	**Without hypertension (*n* = 20,473)**	***P*-value^†^**	**With hypertension (*n* = 47,912)**	**Without hypertension** **(*n* = 12,478)**	***P*-value^‡^**
Age, years	70.0 (62.0–78.0)	72.0 (64.0–79.0)	66.0 (57.0–75.0)	<0.001	71.0 (63.0–78.0)	66.0 (57.0–75.0)	<0.001
Men	32,788 (54.3)	20,997 (52.6)	11,791 (57.6)	<0.001	25,704 (53.6)	7,084 (56.8)	<0.001
BMI, kg/m^2^^*^	24.3 ± 3.9	24.7 ± 3.9	23.4 ± 3.7	<0.001	24.6 ± 3.9	23.1 ± 3.6	<0.001
SBP, mmHg	130.7 ± 21.0	138.0 ± 20.5	116.5 ± 13.1	<0.001	136.0 ± 19.6	110.4 ± 11.5	<0.001
DBP, mmHg	79.3 ± 13.9	82.9 ± 14.4	72.2 ± 9.3	<0.001	82.4 ± 13.5	67.4 ± 7.7	<0.001
Medical insurance				<0.001			<0.001
Urban insurance	36,746 (60.8)	25,534 (64.0)	11,212 (54.8)		29,926 (62.5)	6,820 (54.7)	
Rural insurance	12,499 (20.7)	7,374 (18.5)	5,125 (25.0)		9,405 (19.6)	3,094 (24.8)	
Self-paid	5,062 (8.4)	3,127 (7.8)	1,935 (9.4)		3,840 (8.0)	1,222 (9.8)	
Others	6,083 (10.1)	3,882 (9.7)	2,201 (10.8)		4,741 (9.9)	1,342 (10.8)	
Smoking	12,223 (20.2)	7,826 (19.6)	4,397 (21.5)	<0.001	9,551 (19.9)	2,672 (21.4)	<0.001
Alcohol drinking	7,029 (11.6)	4,549 (11.4)	2,480 (12.1)	<0.001	5,562 (11.6)	1,467 (11.8)	0.646
**Medical history**							
CHD	15,625 (25.9)	11,796 (29.6)	3,829 (18.7)	<0.001	13,286 (27.7)	2,339 (18.7)	<0.001
Heart failure	7,781 (12.9)	5,026 (12.6)	2,755 (13.5)	0.003	5,964 (12.4)	1,817 (14.6)	<0.001
Cerebrovascular disease	8,563 (14.2)	6,660 (16.7)	1,903 (9.3)	<0.001	7,368 (15.4)	1,195 (9.6)	<0.001
Diabetes mellitus	10,088 (16.7)	8,118 (20.3)	1,970 (9.6)	<0.001	8,927 (18.6)	1,161 (9.3)	<0.001
Previous bleeding	986 (1.6)	695 (1.7)	291 (1.4)	0.003	793 (1.7)	193 (1.5)	0.395
**Prehospital treatments**							
Anticoagulant drug	11,397 (18.9)	7,274 (18.2)	4,123 (20.1)	<0.001	8,767 (18.3)	2,630 (21.1)	<0.001
Antiarrhythmic drug	5,042 (8.3)	3,349 (8.4)	1,693 (8.3)	0.612	4,023 (8.4)	1,019 (8.2)	0.408
Antiplatelet drug	13,630 (22.6)	10,038 (25.1)	3,592 (17.5)	<0.001	11,399 (23.8)	2,231 (17.9)	<0.001
Antihypertensive drug	29,223 (48.4)	22,283 (55.8)	6,940 (33.9)	<0.001	24,771 (51.7)	4,452 (35.7)	<0.001
**Procedures/surgery**							
Electrical cardioversion	811 (1.3)	527 (1.3)	284 (1.4)	0.499	630 (1.3)	181 (1.5)	0.241
Catheter ablation	1,986 (3.3)	1,266 (3.2)	720 (3.5)	0.024	1,525 (3.2)	461 (3.7)	0.004
Surgery	203 (0.3)	115 (0.3)	88 (0.4)	0.004	145 (0.3)	58 (0.5)	0.005
**Etiological diagnosis of AF**				<0.001			<0.001
Valvular	8,231 (13.6)	4,271 (10.7)	3,960 (19.3)		5,660 (11.8)	2,571 (20.6)	
Non-valvular	52,159 (86.4)	35,646 (89.3)	16,513 (80.7)		42,252 (88.2)	9,907 (79.4)	
**Types of AF**				<0.001			0.006
First diagnosed	12,417 (20.6)	8,016 (20.1)	4,401 (21.5)		9,750 (20.3)	2,667 (21.4)	
Paroxysmal	23,215 (38.4)	15,523 (38.9)	7,692 (37.6)		18,569 (38.8)	4,646 (37.2)	
Persistent	15,117 (25.0)	9,988 (25.0)	5,129 (25.1)		11,938 (24.9)	3,179 (25.5)	
Permanent	9,641 (16.0)	6,390 (16.0)	3,251 (15.9)		7,655 (16.0)	1,986 (15.9)	
AF as principal discharge diagnosis	24,969 (41.3)	16,056 (40.2)	8,913 (43.5)	<0.001	19,691 (41.1)	5,278 (42.3)	0.015

Values are expressed in median (interquartile range), mean ± standard deviation, or n (%).

ACC/AHA, American College of Cardiology/American Heart Association; BMI, body mass index; SBP, systolic blood pressure; DBP, diastolic blood pressure; CHD, coronary heart disease; AF, atrial fibrillation.

* BMI was not available for 44.5% (26,878/60,390) patients hospitalized with AF, including 14,209 men and 12,669 women.

† *P*-values for comparisons between groups with and without hypertension based on 2018 Chinese hypertension guidelines.

‡ *P*-values for comparisons between groups with and without hypertension based on 2017 ACC/AHA hypertension guidelines.

### Prevalence of hypertension in patients hospitalized with AF

**Figure 1:**
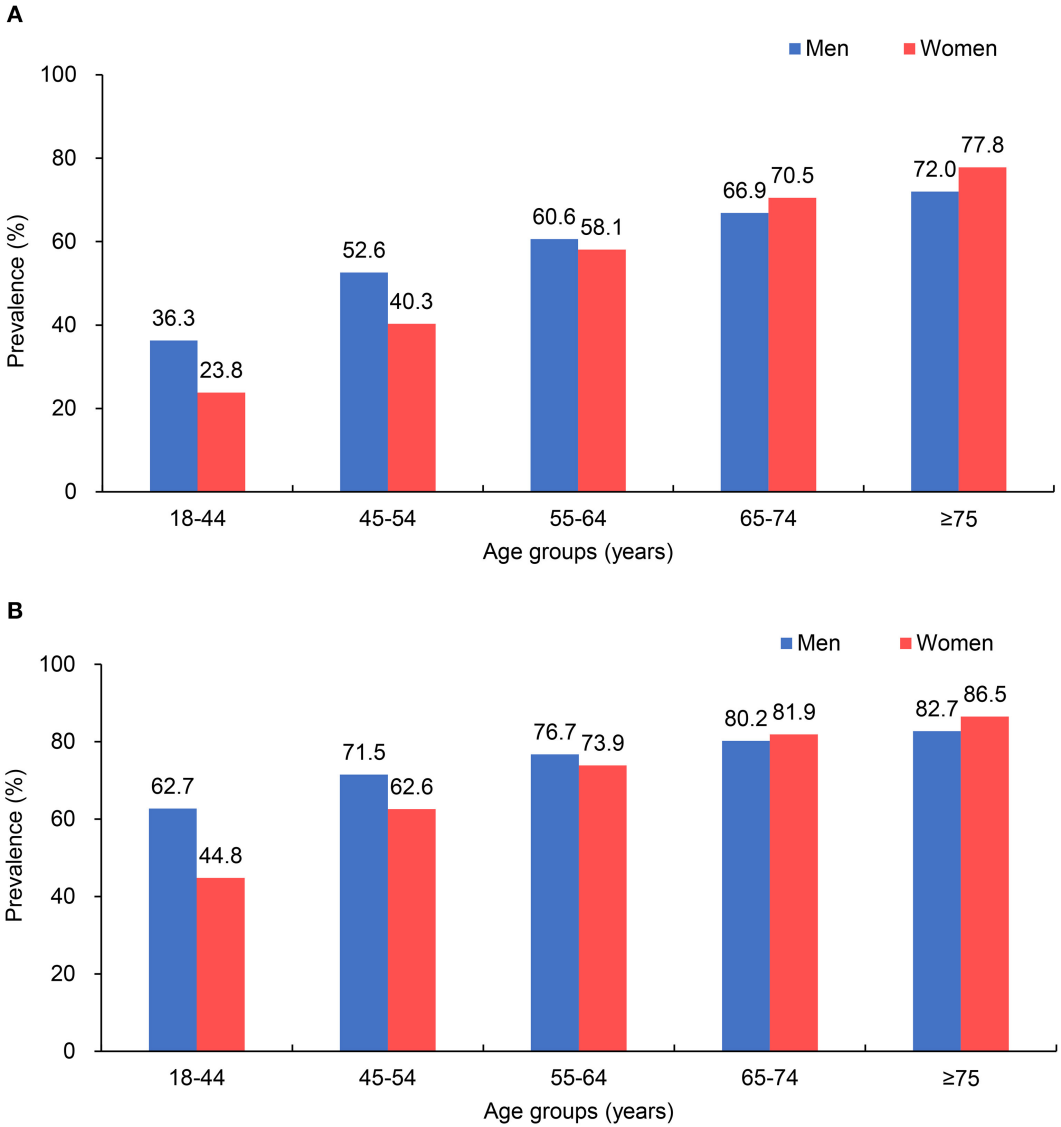
Prevalence of hypertension in patients hospitalized with atrial fibrillation (AF) in different age groups. **(A)** According to the 2018 Chinese hypertension guidelines; **(B)** according to the 2017 American College of Cardiology/American Heart Association (ACC/AHA) hypertension guidelines.

**Figure 2:**
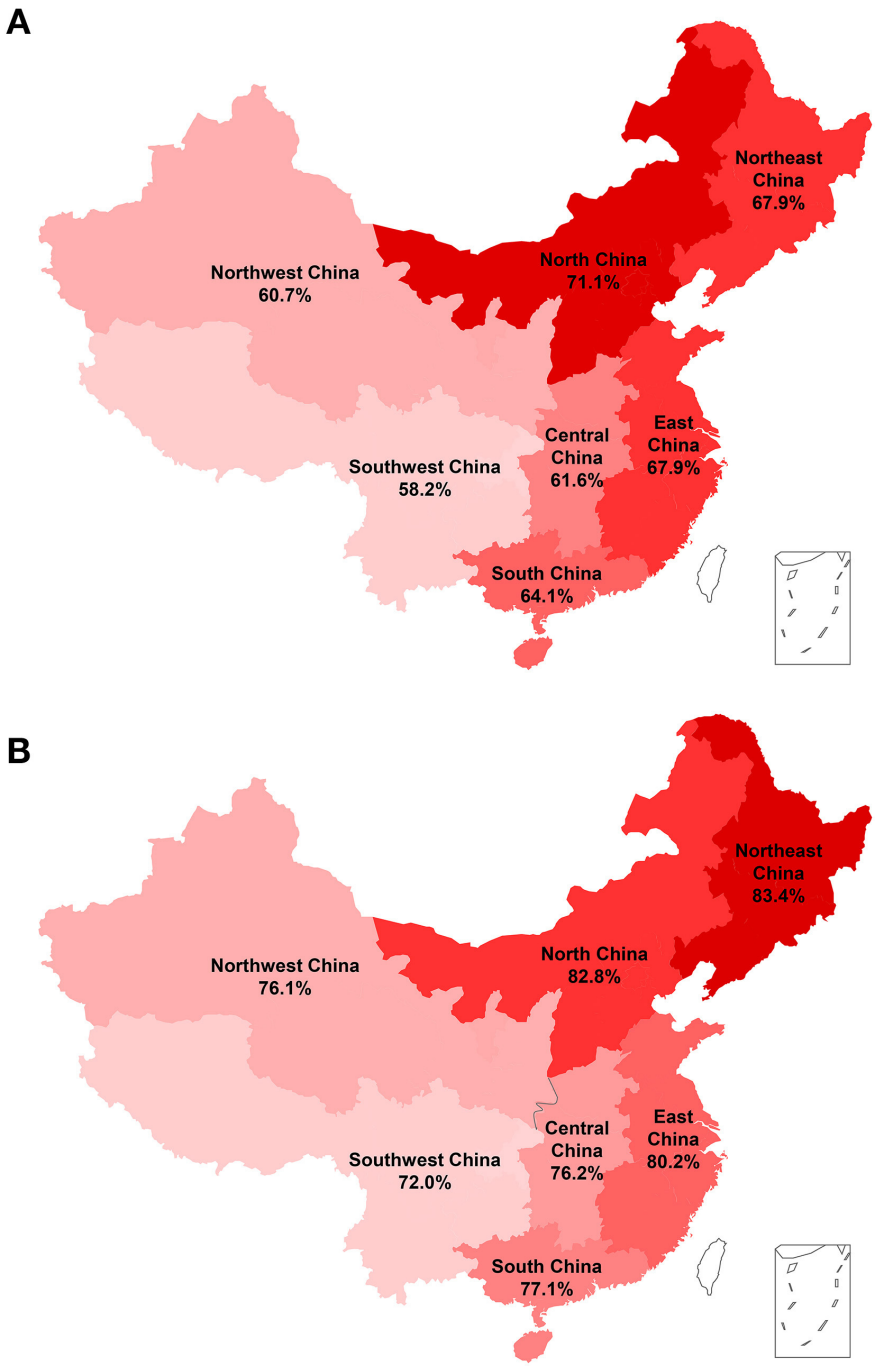
Prevalence of hypertension in patients hospitalized with atrial fibrillation (AF) in seven regions of China. **(A)** According to the 2018 Chinese hypertension guidelines; **(B)** according to the 2017 American College of Cardiology/American Heart Association (ACC/AHA) hypertension guidelines. The prevalence of hypertension is represented in red.

The prevalence of hypertension was 66.1% (39,917/60,390) according to the Chinese diagnostic criteria. A higher prevalence of hypertension was observed in patients with older age, female sex, obesity, urban insurance, and non-valvular AF (Table 3). The prevalence of hypertension was higher in men than in women among patients <65 years of age, but this result was reversed in patients above 65 years of age under either Chinese or ACC/AHA diagnostic criteria (Figure 1). The geographic distribution of the prevalence of hypertension, with a high prevalence in the east and low prevalence in the west, are depicted in Figure 2A; the highest prevalence of hypertension was found in North China (71.1%).

**Table 3:** Prevalence, awareness, treatment, and control rates of hypertension in patients hospitalized with atrial fibrillation, according to the 2018 Chinese and the 2017 ACC/AHA hypertension guidelines.

	**2018 Chinese hypertension guidelines**					**2017 ACC/AHA hypertension guidelines**		
**Characteristics**	**Prevalence**	**Awareness rate**	**Treatment rate**	**Control rate**	**Treatment control rate**	**Prevalence**	**Control rate**	**Treatment control rate**
Overall	39,917 (66.1)	32,034 (80.3)	22,283 (55.8)	15,935 (39.9)	10,302 (46.2)	47,912 (79.3)	8,018 (16.7)	5,255 (21.2)
**Age groups, years**								
18–44	651 (33.5)	330 (50.7)	248 (38.1)	145 (22.3)	92 (37.1)	1,140 (58.6)	58 (5.1)	43 (12.2)
45–54	2,709 (48.6)	1,834 (67.7)	1,345 (49.6)	926 (34.3)	582 (43.3)	3,823 (68.6)	431 (11.3)	285 (17.4)
55–64	7,490 (59.6)	5,761 (76.9)	3,957 (52.8)	2,962 (39.5)	1,907 (48.2)	9,497 (75.6)	1,408 (14.8)	928 (20.3)
65–74	12,737 (68.6)	10,404 (81.7)	7,136 (56.0)	5,124 (40.2)	3,288 (46.1)	15,031 (81.0)	2,522 (16.8)	1,622 (20.5)
≥75	16,330 (75.1)	13,705 (83.9)	9,597 (58.8)	6,778 (41.5)	4,433 (46.2)	18,421 (84.7)	3,599 (19.5)	2,377 (23.1)
**Sex**								
Men	20,997 (64.0)	16,599 (79.1)	11,464 (54.6)	8,344 (39.7)	5,331 (46.5)	25,704 (78.4)	4,214 (16.4)	2,749 (21.4)
Women	18,920 (68.5)	15,435 (81.6)	10,819 (57.2)	7,591 (40.1)	4,971 (45.9)	22,208 (80.5)	3,804 (17.1)	2,506 (21.1)
BMI groups, kg/m^2^^*^								
<18.5	916 (50.1)	671 (73.3)	484 (52.8)	360 (39.3)	222 (45.9)	1,209 (66.1)	191 (15.8)	118 (20.4)
18.5–23.9	8,773 (60.9)	6,781 (77.3)	4,825 (55.0)	3,541 (40.4)	2,285 (47.4)	10,866 (75.4)	1,848 (17.0)	1,184 (21.5)
24.0–27.9	8,697 (70.3)	7,179 (82.5)	5,112 (58.8)	3,599 (41.4)	2,383 (46.6)	10,261 (82.9)	1,745 (17.0)	1,181 (21.1)
≥28.0	3,844 (78.5)	3,278 (85.3)	2,438 (63.4)	1,541 (40.1)	1,093 (44.8)	4,357 (89.0)	701 (16.1)	518 (19.9)
**Medical insurance**								
Urban insurance	25,534 (69.5)	21,138 (82.8)	15,137 (59.3)	10,678 (41.8)	7,167 (47.3)	29,926 (81.4)	5,442 (18.2)	3,701 (22.3)
Rural insurance	7,374 (59.0)	5,332 (72.3)	3,284 (44.5)	2,360 (32.0)	1,239 (37.7)	9,405 (75.2)	1,097 (11.7)	565 (14.5)
Self-paid	3,127 (61.8)	2,479 (79.3)	1,674 (53.5)	1,317 (42.1)	828 (49.5)	3,840 (75.9)	675 (17.6)	433 (23.3)
Others	3,882 (63.8)	3,085 (79.5)	2,188 (56.4)	1,580 (40.7)	1,068 (48.8)	4,741 (77.9)	804 (17.0)	556 (22.7)
**Smoking**								
Yes	7,826 (64.0)	6,107 (78.0)	4,232 (54.1)	3,030 (38.7)	1,915 (45.3)	9,551 (78.1)	1,519 (15.9)	984 (20.8)
No	32,091 (66.6)	25,927 (80.8)	18,051 (56.2)	12,905 (40.2)	8,387 (46.5)	38,361 (79.6)	6,499 (16.9)	4,271 (21.3)
**Alcohol drinking**								
Yes	4,549 (64.7)	3,535 (77.7)	2,485 (54.6)	1,711 (37.6)	1,123 (45.2)	5,562 (79.1)	842 (15.1)	556 (19.9)
No	35,368 (66.3)	28,499 (80.6)	19,798 (56.0)	14,224 (40.2)	9,179 (46.4)	42,350 (79.4)	7,176 (16.9)	4,699 (21.4)
**Etiological diagnosis of AF**								
Valvular	4,271 (51.9)	2,974 (69.6)	2,349 (55.0)	1,471 (34.4)	959 (40.8)	5,660 (68.8)	781 (13.8)	533 (18.2)
Non-valvular	35,646 (68.3)	29,060 (81.5)	19,934 (55.9)	14,464 (40.6)	9,343 (46.9)	42,252 (81.0)	7,237 (17.1)	4,722 (21.6)
**Types of AF**								
First diagnosed	8,016 (64.6)	6,016 (75.0)	3,002 (37.5)	2,814 (35.1)	1,342 (44.7)	9,750 (78.5)	1,440 (14.8)	693 (21.2)
Paroxysmal	15,523 (66.9)	12,829 (82.6)	9,062 (58.4)	6,583 (42.4)	4,385 (48.4)	18,569 (80.0)	3,349 (18.0)	2,274 (22.9)
Persistent	9,988 (66.1)	8,099 (81.1)	6,101 (61.1)	4,093 (41.0)	2,830 (46.4)	11,938 (79.0)	1,995 (16.7)	1,395 (20.3)
Permanent	6,390 (66.3)	5,090 (79.7)	4,118 (64.4)	2,445 (38.3)	1,745 (42.4)	7,655 (79.4)	1,234 (16.1)	893 (19.0)

Values are expressed in n (%).

ACC/AHA, American College of Cardiology/American Heart Association; BMI, body mass index; AF, atrial fibrillation.

* BMI was not available for 44.3% (17,687/39,917) patients hospitalized with AF and hypertension based on the 2018 Chinese hypertension guidelines; BMI was not available for 44.3% (21,219/47,912) patients hospitalized with AF and hypertension based on the 2017 ACC/AHA hypertension guidelines.

The prevalence of hypertension was 79.3% based on the ACC/AHA diagnostic criteria, and similar distribution patterns were found in different patient groups and geographical regions compared to those based on the Chinese diagnostic criteria (Table 3; Figure 2B); however, Northeast China had the highest prevalence (83.4%).

### Awareness, treatment, and control rates of hypertension in patients hospitalized with AF

According to the Chinese diagnostic criteria, the awareness, treatment and control rates of hypertension in patients were 80.3, 55.8 and 39.9%, respectively (Table 3). These rates were lower in younger patients, men, underweight patients, those with rural insurance, those who smoked, those who consumed alcohol, patients with valvular AF, and first diagnosed AF patients. Among patients treated for hypertension, only 46.2% (10,302/22,283) had well-controlled BP. The treatment control rate was lower in the younger patients, women, obese patients, those with rural insurance, those who smoked, those who consumed alcohol, patients with valvular AF and patients with permanent AF. In patients with concomitant diseases, including CHD, heart failure, and diabetes mellitus, the control and treatment control rates of hypertension were <50 and 25%, respectively, with treatment goals of 140/90 and 130/80 mm Hg (Supplementary Table 5). Compared with other regions, the awareness (75.0%), control (31.5%) and treatment control rates (37.9%) in Northeast China were the lowest (Supplementary Figure 2B).

Both the control rate (16.7%) and treatment control rate (21.2%) according to the ACC/AHA diagnostic criteria were much lower than that based on the Chinese diagnostic criteria (Table 3). However, the distribution of patient groups and geographic regions was similar to that using Chinese diagnostic criteria (Table 3; Supplementary Figure 2C).

### Trends in the hypertensive status of patients hospitalized with AF from 2015 to 2019

**Figure 3:**
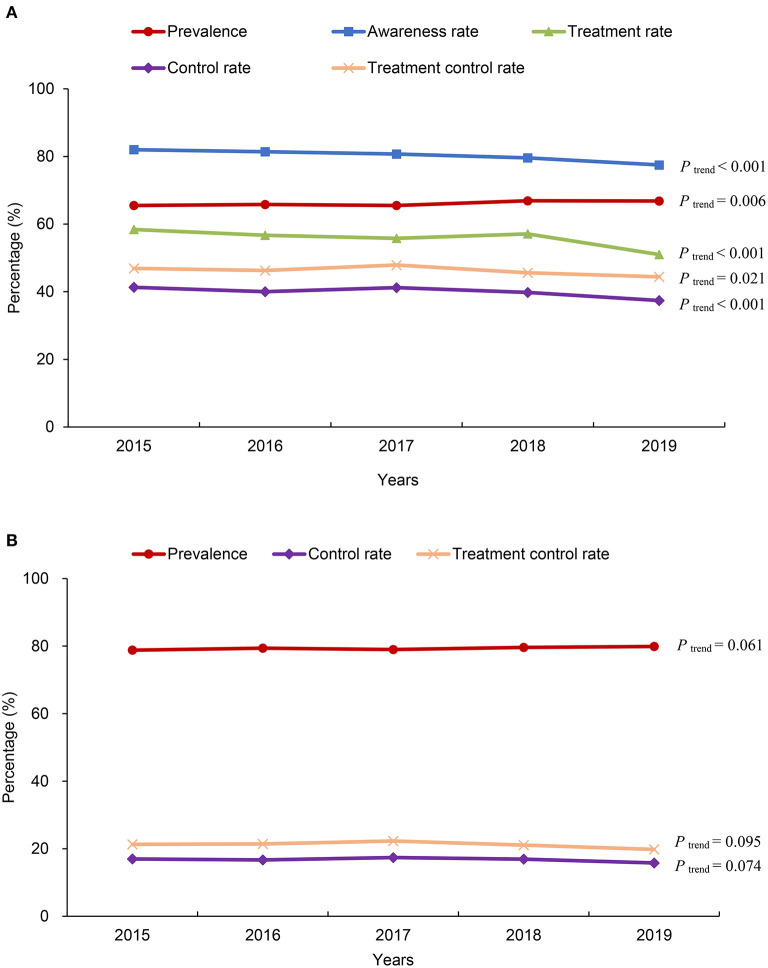
Changes in the status of hypertension in patients hospitalized with atrial fibrillation (AF) in China from 2015 to 2019. **(A)** According to the 2018 Chinese hypertension guidelines; **(B)** according to the 2017 American College of Cardiology/American Heart Association (ACC/AHA) hypertension guidelines.

The prevalence of hypertension in patients hospitalized with AF increased from 65.5% in 2015 to 66.8% in 2019, showing an upward trend (*P* for trend = 0.006), based on the Chinese diagnostic criteria. Moreover, decreasing trends were found for the rates of awareness, treatment, control and treatment control (all *Ps* for trend < 0.05) (Figure 3A). When the ACC/AHA diagnostic criteria were used, the trends in the prevalence, control rate and treatment control rate of hypertension did not reach statistical significance (Figure 3B).

### Prehospital use of antihypertensive drugs in hypertensive patients hospitalized with AF receiving antihypertensive therapy

The most frequently used antihypertensive drugs among patients on BP-lowering drugs prior to their current admission were β blockers (55.9%), followed by CCBs (34.5%) and diuretics (25.1%). Renin-angiotensin-aldosterone system (RAAS) inhibitory drugs (ACEIs/ARBs) were used by 41.9% of the patients. Overall, 51.8, 31.0, and 17.2% of the treated patients were taking 1 drug, 2 drugs, and ≥3 drugs, respectively. Among the patients with BP ≥ 160/100 mm Hg, 51.2% were taking only 1 type of antihypertensive drug (Supplementary Table 6).

### Factors associated with uncontrolled hypertension in patients hospitalized with AF

**Figure 4:**
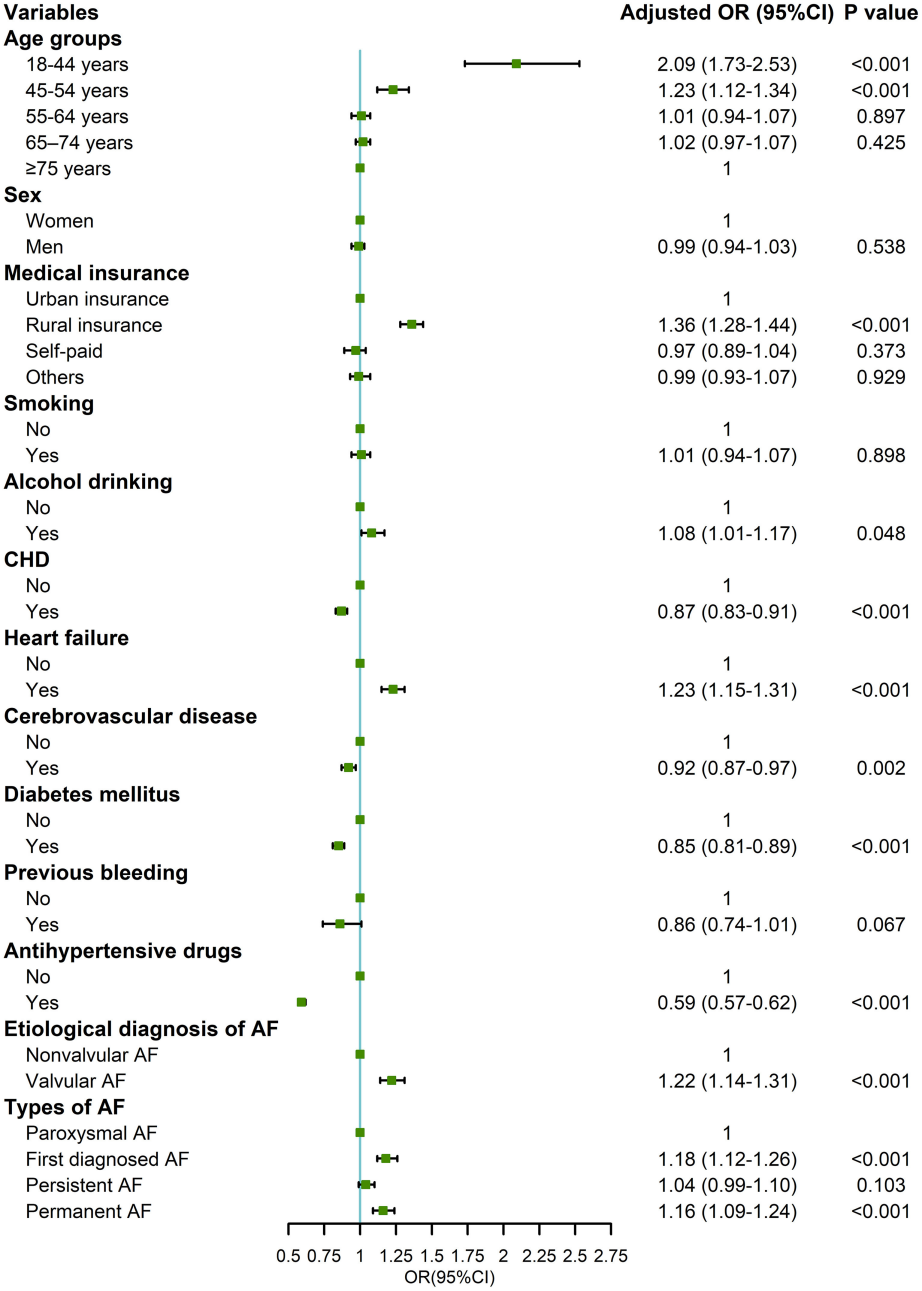
Factors associated with uncontrolled hypertension in patients hospitalized with atrial fibrillation and hypertension based on the 2018 Chinese hypertension guidelines. OR, odds ratio; CI, confidence interval; CHD, coronary heart disease; AF, atrial fibrillation. Adjusted variables: age, sex, medical insurance, smoking, alcohol drinking, CHD, heart failure, cerebrovascular disease, diabetes mellitus, previous bleeding, antihypertensive drugs, etiological diagnosis of AF, and types of AF.

In the two-level logistic regression analysis, factors associated with uncontrolled hypertension included young patients [18–44 and 45–54 years old compared with ≥75 years old, odds ratio (OR) = 2.09, 95% confidence interval (CI): 1.73–2.53 and 1.23, 95% CI: 1.12–1.34, respectively], rural insurance (OR = 1.36, 95% CI: 1.28–1.44, compared with urban insurance), alcohol drinking (OR = 1.08, 95% CI: 1.01–1.17), history of heart failure (OR = 1.23, 95% CI: 1.15–1.31), valvular AF (OR = 1.22, 95% CI: 1.14–1.31), first diagnosed and permanent AF (OR = 1.18, 95% CI: 1.12–1.26 and OR = 1.16, 95% CI: 1.09–1.24, respectively, compared with paroxysmal AF), according to the Chinese diagnostic criteria (Figure 4). Similar results were also observed based on the ACC/AHA diagnostic criteria (Supplementary Table 7) and in the sensitivity analysis of hypertensive patients hospitalized with AF and for which BMI data was obtained (Supplementary Table 8).

## Discussion

To the best of our knowledge, this is the first study to focus on the status of hypertension in patients hospitalized with AF in China. Our study provided comprehensive and up-to-date data. We found that 66.1 and 79.3% of patients hospitalized with AF had hypertension using cutoff values of 140/90 and 130/80 mm Hg, respectively. Although the majority of hypertensive patients were aware of their condition, the treatment and control rates of this condition were rather low and varied greatly among different patient groups and geographic regions. Factors associated with uncontrolled BP were identified to guide improvement in measure of BP control in patients with AF in China.

Although a few studies have reported a prevalence of hypertension in China ranging from 55.5 to 72.5% as the clinical characteristics in patients with AF (10–13), those studies were conducted a decade ago or had a small sample size. Our study adopted the method of stratified sampling at geographic and economic levels and obtained a large, nationally representative sample. When 140/90 mm Hg was used as the cutoff value of hypertension, the prevalence of hypertension in our study (66.1%) was lower than that in a sub-study of the Rivaroxaban Once Daily Oral Direct Factor Xa Inhibition Compared with Vitamin K Antagonism for Prevention of Stroke and Embolism Trial in Atrial Fibrillation (ROCKET AF) (90.5%) (5) and that in the Outcomes Registry for Better Informed Treatment of Atrial Fibrillation (ORBIT-AF) (83.3%) (6), but similar to that in a sub-study of the Randomized Evaluation of Long-Term Anticoagulation Therapy (RE-LY) AF registry (66.5%) (7) and that in the Korean National Health Insurance Service database (62.2%) (8). Although the prevalence of hypertension varies by ethnicity, age, and patient risk, the above results indicate that patients with AF face a high burden of hypertension worldwide.

Although hypertension is associated with poor prognosis in patients with AF, limited data on the awareness and treatment of hypertension in patients with AF are available in China. Our study showed that hypertensive patients hospitalized with AF had a high awareness rate of hypertension, but the treatment rate was still unsatisfactory. Compared with the hypertension control rate in the general population, the hypertension control rate in patients with AF seemed to be better (23). A possible explanation is that patients with AF have more opportunities to obtain advice from their doctors and have better treatment compliance. The lower awareness of hypertensive status in patients with their first diagnosed AF also supports this notion. However, from the perspective of improving the prognosis of patients with AF, there is vast room for improvement in the treatment and control rates of hypertension in China. The control rate of hypertension in our study was much lower than those in international multicenter or national registry studies (5–9). Even in patients receiving antihypertensive treatment, more than half did not reach the treatment target. More importantly, decreasing trends were observed for the awareness, treatment, control, and treatment control rates of hypertension between 2015 and 2019.

To identify the obstacles in the management of hypertension in patients with AF and to guide strategies to improve quality in practice, we further analyzed the factors associated with uncontrolled hypertension. We found that patients who were younger, had rural insurance, consumed alcohol or who were first diagnosed AF were less likely to have controlled BP. The possible explanations include low health awareness or poor compliance with regular treatment in these patient groups, which may reduce the effect of antihypertensive treatment, especially for patients that consume alcohol (24). Patients with heart failure, valvular AF, and permanent AF usually have complex comorbidities, which may increase the difficulty of BP control. These results indicate that more attention should be given to hypertension management strategies for patients with AF and the above clinical characteristics.

Our findings have some important clinical implications. First, hypertensive patients with AF should have a standardized BP management strategy to reduce the risk of embolism and bleeding, which is the class I recommendation of the current consensus (17). However, the control rate of hypertension was not satisfactory, and 15% of AF patients had a BP ≥ 160/100 mm Hg. Moreover, from 2015 to 2019, the prevalence of hypertension in patients with AF increased while the control rate decreased. The task of prevention, screening and control of hypertension in patients with AF in China is arduous; therefore, effective management measures are necessary and urgent. Second, our study identified vulnerable patient groups and geographic areas that had worse management of hypertension. The large regional variations imply that regional living habits and quality of care should be improved in specific areas, especially in Northeast China. Third, when patient BP exceeds 160/100 mm Hg, combination therapy with ≥ 2 first-line antihypertensive drugs is recommended by the Chinese guidelines for hypertension (20). However, more than half of patients were receiving monotherapy, suggesting that there is still much room for improvement in the management of hypertension in future clinical practice. Fourth, after considering the factors associated with uncontrolled hypertension, unhealthy lifestyle changes, BP management education, and new methods and technology that can improve BP control may be urgently needed (25–27).

Our study has a few limitations. First, this study was retrospective, and BP values in patients with AF were extracted from medical records. Although we adopted third-party monitoring to ensure the accuracy of information extracted from medical records, the accuracy of physician measurements and chart records could not be evaluated. Second, information on pre-hospital antihypertensive drugs may be subject to recall bias. Third, the models of sphygmomanometer instruments may vary among the included hospitals during the study period, although all instruments should have been calibrated as required by regulation authorities. Given that BP measurement methods specifically for patients with AF have not been developed in clinical practice in the past, the accuracy of measuring BP by oscillometric methods in patients with AF was still lower than that in patients with sinus rhythm. Finally, hypertension was defined based on admission BP measurements in our study rather than 3 or more measurements on different days as recommended by current guidelines, which may lead to some information bias.

## Conclusion

Hypertension is common in hospitalized Chinese patients with AF. Although most patients are aware of this comorbidity, the treatment and control rates of hypertension are currently unsatisfactory, and variations in these rates according to patient population and geographic area exist. Therefore, it is urgent to implement effective measures to improve the prevention and management of hypertension and reduce inequality in patients with AF in China.

## Data availability statement

The original contributions presented in the study are included in the article/Supplementary material, further inquiries can be directed to the corresponding authors.

## Ethics statement

The studies involving human participants were reviewed and approved by Ethics Committee of Beijing Anzhen Hospital, Capital Medical University. Written informed consent for participation was not required for this study in accordance with the national legislation and the institutional requirements.

## Author contributions

The study was conceived and designed by JiL and CS. The data was collected and interpreted by DZ, JiL, JuL, YH, NY, YQ, DH, YY, and HW. The data were analyzed and the first draft of the manuscript was prepared by ZS. All authors critically revised the manuscript for important intellectual content, read, and approved the final manuscript.

## Funding

The CCC-AF project was supported by a collaborative project of the American Heart Association (AHA) and the Chinese Society of Cardiology. The AHA received funding from Pfizer through an independent grant for learning and change and AstraZeneca as a quality improvement initiative. Part of the work was supported by an unrestricted grant from Boehringer-Ingelheim (China). Boehringer-Ingelheim (China) was not involved in the study design, collection, analysis, interpretation of data, the writing of this article or the decision to submit it for publication.

## Conflict of interest

The authors declare that the research was conducted in the absence of any commercial or financial relationships that could be construed as a potential conflict of interest.

## Publisher's note

All claims expressed in this article are solely those of the authors and do not necessarily represent those of their affiliated organizations, or those of the publisher, the editors and the reviewers. Any product that may be evaluated in this article, or claim that may be made by its manufacturer, is not guaranteed or endorsed by the publisher.
