# Supplementary material for: Prevalence, awareness, treatment, and control rates of hypertension in patients hospitalized with atrial fibrillation in China: Findings from the CCC-AF project
Source: Front Cardiovasc Med. 2022 Aug 1;9:970787. doi: 10.3389/fcvm.2022.970787 (PMC9376459; doi:10.3389/fcvm.2022.970787)
Supplement: Supplementary file 1 [file Data_Sheet_1.doc]

**SUPPLEMENTAL MATERIAL**

**Table 1.** Hospital sampling frame of the Improving Care for Cardiovascular Disease in China-Atrial Fibrillation (CCC-AF) project

**Table 2.** Definition of study variables

**Table 3.** Variable assignment of multivariable analysis of logistic regression

**Table 4.** Missing rates of study variables and management of missing data

**Table 5.** Control of hypertension in patients hospitalized with atrial fibrillation (AF) and other comorbidities using different goals of treatment, based on the 2018 Chinese hypertension guidelines

**Table 6.** Prehospital use of antihypertensive drugs in patients hospitalized with atrial fibrillation (AF) and hypertension receiving antihypertensive therapy

**Table 7.** Factors associated with uncontrolled hypertension in patients hospitalized with atrial fibrillation (AF) and hypertension based on the 2017 American College of Cardiology/American Heart Association (ACC/AHA) hypertension guidelines

**Table 8.** Factors associated with uncontrolled hypertension in patients hospitalized with atrial fibrillation (AF) and hypertension with body mass index (BMI) data

**Table 9.** Investigators of the Improving Care for Cardiovascular Disease in China-Atrial Fibrillation (CCC-AF) project

**Figure 1.** Proportions of patients hospitalized with atrial fibrillation (AF) with different blood pressure levels in China.

**Figure 2.** Awareness, treatment, and control rates of hypertension in patients hospitalized with atrial fibrillation (AF) in different regions of China.

**Table 1. Hospital sampling frame of the Improving Care for Cardiovascular Disease in China-Atrial Fibrillation (CCC-AF) project**

| **Regions** | **Economic level*** | **Provinces** | **No. of hospitals in the area**† | **No. of hospitals needed**‡ | **Enrolled** |
| --- | --- | --- | --- | --- | --- |
| **North**  **China** | Low | NA | - | - | - |
|  | Medium-low | Shanxi | 305 | 9 | 10 |
|  | Medium-high | Hebei | 437 | 11 | 15 |
|  | High | Beijing, Tianjin, Inner Mongolia | 434 | 18 | 16 |
| **Northeast**  **China** | Low | NA | - | - | - |
|  | Medium-low | Heilongjiang | 378 | 12 | 12 |
|  | Medium-high | Jilin | 212 | 7 | 5 |
|  | High | Liaoning | 318 | 14 | 13 |
| **East**  **China** | Low | Anhui, Jiangxi | 463 | 14 | 10 |
|  | Medium-low | NA | - | - | - |
|  | Medium-high | Fujian, Shandong | 600 | 20 | 19 |
|  | High | Shanghai, Jiangsu, Zhejiang | 739 | 31 | 32 |
| **Central**  **China** | Low | NA | - | - | - |
|  | Medium-low | Henan, Hunan | 782 | 24 | 27 |
|  | Medium-high | Hubei | 274 | 10 | 7 |
|  | High | NA | - | - | - |
| **South China** | Low | Guangxi | 227 | 8 | 7 |
|  | Medium-low | Hainan | 35 | 1 | 3 |
|  | Medium-high | NA | - | - | - |
|  | High | Guangdong | 385 | 15 | 13 |
| **Southwest China** | Low | Guizhou, Yunnan, Xizang | 471 | 15 | 11 |
|  | Medium-low | Sichuan | 434 | 14 | 13 |
|  | Medium-high | Chongqing | 120 | 4 | 6 |
|  | High | NA | - | - | - |
| **Northwest China** | Low | Gansu | 191 | 6 | 4 |
|  | Medium-low | Qinghai, Xinjiang | 300 | 7 | 8 |
|  | Medium-high | Shaanxi, Ningxia | 354 | 10 | 5 |
|  | High | NA | - | - | - |
| **Total** |  | | **7459** | **250** | **236** |

* Economic level is from National Bauru of Statistical, provinces are grouped into quadruplets according to GDP per capital, low:<29 608.00 RMB, medium-low: 29 608.00-36 393.00 RMB, medium-high: 36 394.00-54 095.00 RMB, high: >54 095.00 RMB.

† Numbers of hospitals are from China Statistical Yearbook 2013.

‡ 10 % for tertiary hospitals and 1.7% for secondary hospitals.

**Table 2.** **Definition of study variables**

| **Variables** | **Definition** |
| --- | --- |
| **Medical insurance** |  |
| Urban insurance | Urban Employees Basic Medical Insurance  Urban Residents Basic Medical Insurance |
| Rural insurance | New Rural Cooperative Medical Insurance |
| Self-paid | No medical insurance |
| Others | Special assistance for the poor  Commercial insurance  Full reimbursement  Other types of medical insurance |
| **Smoking** | Current smoking or smoking cessation within 1 year |
| **Medical history** |  |
| CHD | Previous history of CHD or having received PCI before this hospitalization. |
| Heart failure | Previously history of heart failure |
| Cerebrovascular disease | Previously history of ischemic stroke, hemorrhagic stroke, TIA or other related diseases |
| Diabetes mellitus | Previously history of diabetes mellitus |
| Previous bleeding | Previously history of previous bleeding |
| **Prehospital treatments** |  |
| Anticoagulant drug  Antiarrhythmic drug  Antiplatelet drug  Antihypertensive drug | Prehospital treatments were defined based on the original admission records |
| **Procedures/Surgery** |  |
| Electrical cardioversion  Catheter ablation  Surgery | Operation/surgery were defined based on the original admission records |
| **Etiology classification of AF** |  |
| Valvular | Valvular AF specifically referred to AF combined after mechanical valve replacement and in patients with moderate or severe mitral stenosis (1) |
| Nonvalvular | Patients with AF other than valvular AF |
| **Types of AF** |  |
| First diagnosed | Types of AF were defined based on the original admission records |
| Paroxysmal |
| Persistent |
| Permanent |
| **AF as principal discharge diagnosis** | Based on the principal discharge diagnosis records |

Abbreviations: CHD, Coronary heart disease; PCI, percutaneous coronary intervention; TIA, transient ischemic attacks, AF, atrial fibrillation.

**References**

1. Huang C, Zhang S, Hua W. Current Knowledge and Management of Atrial Fibrillation: Consensus of Chinese Experts 2021*. Chin J Cardiac Arrhy*th. (2022) 26(1):15-88. Epub 2022/02. doi: 10.3760 / cma.j.cn113859-20211224-00264.

**Table 3. Variable assignment of multivariable analysis of logistic regression**

| **Factors** | **Assignment** |
| --- | --- |
| Age groups | 1= ≥75 years, 2= 18-44 years, 3= 45-54 years, 4= 55-64 years,  5= 65-74 years |
| Sex | 0=Women, 1=Men |
| BMI groups | 1=18.5–23.9 kg/m², 2=<18.5 kg/m², 3=24.0–27.9 kg/m²,  4=≥28.0 kg/m² |
| Medical insurance | 1= Urban insurance, 2= Rural insurance, 3= Self-paid, 4= Others |
| Smoking | 0=No, 1=Yes |
| Alcohol drinking | 0=No, 1=Yes |
| CHD | 0=No, 1=Yes |
| Heart failure | 0=No, 1=Yes |
| Cerebrovascular disease | 0=No, 1=Yes |
| Diabetes mellitus | 0=No, 1=Yes |
| Previous bleeding | 0=No, 1=Yes |
| Antihypertensive drug | 0=No, 1=Yes |
| Etiological diagnosis of AF | 0= Nonvalvular, 1= Valvular |
| Types of AF | 1= Paroxysmal, 2= First diagnosed, 3= Persistent, 4= Permanent |
| Hypertension control | 0= Controlled, 1= Uncontrolled |

Abbreviations: BMI, body mass index; CHD, coronary heart disease; AF, atrial fibrillation.

**Table** **4. Missing rates of study** **variables and management of missing data^*^**

| **Variables** | **Missing data, % (No.)** | **Management of missing data** |
| --- | --- | --- |
| Age | 0.08% (47/60 390) | Imputed using sequential regression multiple imputation method implemented by IVE ware software |
| Medical insurance | 0.00% (3/60 390) |
| Etiological diagnosis of AF | 0.11% (67/60 390) |
| Types of AF | 0.14% (85/60 390) |

^*^ Variables without any missing were not list in the following table.

Abbreviations: AF, atrial fibrillation.

**Table 5. Control of hypertension in patients hospitalized with atrial fibrillation (AF) and other comorbidities using different goals of treatment, based on the 2018 Chinese hypertension guidelines**

| **Comorbidities** | **Control rate**^*^ | **Treatment control rate**^*^ | **Control rate†** | **Treatment control rate†** |
| --- | --- | --- | --- | --- |
| CHD | 5110 (43.3) | 3489 (46.8) | 2688 (22.8) | 1857 (24.9) |
| Heart failure | 1861 (37.0) | 1418 (40.7) | 990 (19.7) | 760 (21.8) |
| Diabetes mellitus | 3661 (45.1) | 2540 (48.0) | 1914 (23.6) | 1336 (25.3) |

Values are expressed in n (%).

Abbreviations: CHD, coronary heart disease; SBP, systolic blood pressure; DBP, diastolic blood pressure.

* The goal was 140 mm Hg for SBP and 90 mm Hg for DBP.

† The goal was 130 mm Hg for SBP and 80 mm Hg for DBP.

**Table 6.** **Prehospital use of antihypertensive drugs in patients hospitalized with atrial fibrillation and hypertension** **receiving antihypertensive therapy***

| **Variables** | **Total**  **(n=22 283)** | **Blood pressure levels at admission (mm Hg)** | | | |
| --- | --- | --- | --- | --- | --- |
|  |  | **<130/<80**  **(n=5255)** | **130-139/80-89**  **(n=5047)** | **140-159/90-99**  **(n=7517)** | **≥160/100**  **(n=4464)** |
| **Drug Type** |  |  |  |  |  |
| β blockers | 12 448 (55.9) | 2897 (55.1) | 2826 (56.0) | 4366 (58.1) | 2359 (52.8) |
| CCBs | 7689 (34.5) | 1769 (33.7) | 1932 (38.3) | 2362 (31.4) | 1635 (36.6) |
| Diuretics | 5602 (25.1) | 1353 (25.7) | 1093 (21.7) | 1953 (26.0) | 1203 (26.9) |
| ACEIs/ARBs | 9344 (41.9) | 2304 (43.8) | 2161 (42.8) | 2958 (39.4) | 1921 (43.0) |
| ARAs | 2787 (12.5) | 657 (12.5) | 556 (11.0) | 955 (12.7) | 619 (13.9) |
| α blockers | 82 (0.4) | 16 (0.3) | 14 (0.3) | 28 (0.4) | 24 (0.5) |
| Direct-acting vasodilators | 65 (0.3) | 15 (0.3) | 9 (0.2) | 27 (0.4) | 14 (0.3) |
| **Number of drugs for combination therapy**† |  |  |  |  |  |
| 1 | 11 540 (51.8) | 2677 (50.9) | 2597 (51.5) | 3981 (53.0) | 2285 (51.2) |
| 2 | 6901 (31.0) | 1639 (31.2) | 1602 (31.7) | 2312 (30.8) | 1348 (30.2) |
| ≥3 | 3842 (17.2) | 939 (17.9) | 848 (16.8) | 1224 (16.3) | 831 (18.6) |

Values are expressed in n (%).

Abbreviation: ACEIs=angiotensin converting enzyme inhibitors, ARBs=angiotensin receptor blockers, CCBs= calcium channel blockers, ARAs=aldosterone receptor antagonists.

* Hypertension: according to the 2018 Chinese hypertension guidelines.

† “1” for one drug of monotherapy; “2” for two drugs free combinations; “3” for ≥3 drugs free combinations.

**Table 7. Factors associated with uncontrolled hypertension in patients hospitalized with atrial fibrillation (AF) and hypertension based on the 2017 ACC/AHA hypertension guidelines**

| **Variables** | **Unadjusted**  **OR (95%CI)** | ***P* value** | **Adjusted**  **OR (95%CI)** | ***P* value** |
| --- | --- | --- | --- | --- |
| **Age groups**, years |  |  |  |  |
| 18-44 | 4.53 (3.47-5.91) | ＜0.001 | 3.56 (2.72-4.67) | ＜0.001 |
| 45-54 | 1.91 (1.72-2.12) | ＜0.001 | 1.61 (1.44-1.80) | ＜0.001 |
| 55-64 | 1.40 (1.30-1.49) | ＜0.001 | 1.24 (1.16-1.34) | ＜0.001 |
| 65-74 | 1.20 (1.14-1.27) | ＜0.001 | 1.15 (1.08-1.21) | ＜0.001 |
| ≥75 | 1 |  | 1 |  |
| **Sex** |  |  |  |  |
| Men | 1.05 (1.01-1.11) | 0.032 | 0.99 (0.94-1.05) | 0.857 |
| Women | 1 |  | 1 |  |
| **Medical insurance** |  |  |  |  |
| Urban insurance | 1 |  | 1 |  |
| Rural insurance | 1.68 (1.57-1.80) | ＜0.001 | 1.46 (1.36-1.57) | ＜0.001 |
| Self-paid | 1.04 (0.95-1.14) | 0.358 | 0.97 (0.89-1.07) | 0.745 |
| Others | 1.09 (1.01-1.18) | 0.041 | 1.00 (0.92-1.09) | 0.995 |
| **Smoking** | 1.08 (1.02-1.15) | 0.015 | 0.99 (0.92-1.07) | 0.745 |
| **Alcohol drinking** | 1.14 (1.06-1.27) | 0.001 | 1.06 (0.96-1.16) | 0.256 |
| **Medical history** |  |  |  |  |
| CHD | 0.72 (0.68-0.76) | ＜0.001 | 0.82 (0.78-0.87) | ＜0.001 |
| Heart failure | 1.01 (0.94-1.09) | 0.765 | 1.12 (1.04-1.21) | 0.003 |
| Cerebrovascular disease | 0.79 (0.74-0.84) | ＜0.001 | 0.90 (0.84-0.96) | 0.001 |
| Diabetes mellitus | 0.68 (0.64-0.72) | ＜0.001 | 0.79 (0.74-0.84) | ＜0.001 |
| Previous bleeding | 0.65 (0.55-0.77) | ＜0.001 | 0.76 (0.64-0.90) | 0.001 |
| **Antihypertensive drugs** | 0.50 (0.48-0.53) | ＜0.001 | 0.56 (0.53-0.59) | ＜0.001 |
| **Etiological diagnosis of AF** |  |  |  |  |
| Valvular | 1.29 (1.20-1.40) | ＜0.001 | 1.19 (1.09-1.29) | ＜0.001 |
| Nonvalvular | 1 |  | 1 |  |
| **Types of AF** |  |  |  |  |
| Paroxysmal | 1 |  | 1 |  |
| First diagnosed | 1.27 (1.19-1.36) | ＜0.001 | 1.09 (1.02-1.18) | 0.008 |
| Persistent | 1.08 (1.03-1.17) | 0.003 | 1.12 (1.06-1.20) | ＜0.001 |
| Permanent | 1.15 (1.07-1.23) | ＜0.001 | 1.20 (1.11-1.29) | ＜0.001 |

Abbreviations: ACC/AHA, American College of Cardiology/American Heart Association; OR, odds ratio; CI, confidence interval; CHD, coronary heart disease; AF, atrial fibrillation.

Adjusted variables: age, sex, medical insurance, smoking, alcohol drinking, CHD, heart failure, cerebrovascular disease, diabetes mellitus, previous bleeding, antihypertensive drugs, etiological diagnosis of AF, and types of AF.

**Table 8. Factors associated with uncontrolled hypertension in patients hospitalized with atrial fibrillation and hypertension with BMI data**

|  | **2018 Chinese hypertension guidelines** | | | |  | **2017 ACC/AHA hypertension guidelines** | | | |
| --- | --- | --- | --- | --- | --- | --- | --- | --- | --- |
| **Variables** | **Unadjusted**  **OR (95%CI)** | ***P* value** | **Adjusted**  **OR (95%CI)** | ***P* value** |  | **Unadjusted**  **OR (95%CI)** | ***P* value** | **Adjusted**  **OR (95%CI)** | ***P* value** |
| **Age groups**, years |  |  |  |  |  |  |  |  |  |
| 18-44 | 2.38 (1.87-3.04) | ＜0.001 | 2.01 (1.56-2.58) | ＜0.001 |  | 4.05 (2.91-5.64) | ＜0.001 | 3.17 (2.27-4.45) | ＜0.001 |
| 45-54 | 1.34 (1.20-1.50) | ＜0.001 | 1.22 (1.09-1.37) | ＜0.001 |  | 1.82 (1.59-2.09) | ＜0.001 | 1.56 (1.35-1.80) | ＜0.001 |
| 55-64 | 1.10 (1.02-1.18) | 0.015 | 1.01 (0.93-1.10) | 0.771 |  | 1.38 (1.26-1.51) | ＜0.001 | 1.23 (1.12-1.35) | ＜0.001 |
| 65-74 | 1.03 (0.97-1.10) | 0.310 | 0.99 (0.93-1.07) | 0.953 |  | 1.16 (1.07-1.25) | ＜0.001 | 1.10 (1.02-1.19) | 0.015 |
| ≥75 | 1 |  | 1 |  |  | 1 |  | 1 |  |
| **Sex** |  |  |  |  |  |  |  |  |  |
| Men | 0.99 (0.94-1.04) | 0.645 | 0.96 (0.90-1.02) | 0.149 |  | 1.06 (0.99-1.13) | 0.103 | 0.99 (0.93-1.07) | 0.951 |
| Women | 1 |  | 1 |  |  | 1 |  | 1 |  |
| **BMI groups**, kg/m² * |  |  |  |  |  |  |  |  |  |
| <18.5 | 1.05 (0.91-1.20) | 0.533 | 0.99 (0.85-1.14) | 0.848 |  | 1.09 (0.93-1.29) | 0.287 | 1.07 (0.90-1.26) | 0.452 |
| 18.5–23.9 | 1 |  | 1 |  |  | 1 |  | 1 |  |
| 24.0–27.9 | 0.96 (0.90-1.02) | 0.170 | 0.99 (0.93-1.06) | 0.854 |  | 1.00 (0.93-1.08) | 0.998 | 1.02 (0.95-1.10) | 0.623 |
| ≥28.0 | 1.01 (0.94-1.09) | 0.773 | 1.06 (0.97-1.15) | 0.180 |  | 1.07 (0.97-1.18) | 0.170 | 1.09 (0.99-1.21) | 0.071 |
| **Medical insurance** |  |  |  |  |  |  |  |  |  |
| Urban insurance | 1 |  | 1 |  |  | 1 |  | 1 |  |
| Rural insurance | 1.49 (1.38-1.61) | ＜0.001 | 1.32 (1.22-1.43) | ＜0.001 |  | 1.65 (1.50-1.81) | ＜0.001 | 1.44 (1.31-1.59) | ＜0.001 |
| Self-paid | 1.09 (0.98-1.21) | 0.105 | 1.07 (0.97-1.20) | 0.190 |  | 1.21 (1.07-1.38) | 0.003 | 1.15 (1.01-1.31) | 0.038 |
| Others | 1.05 (0.96-1.15) | 0.337 | 1.02 (0.93-1.12) | 0.651 |  | 1.06 (0.95-1.18) | 0.318 | 1.01 (0.91-1.13) | 0.818 |
| **Smoking** | 1.05 (0.98-1.12) | 0.138 | 1.02 (0.94-1.10) | 0.662 |  | 1.09 (1.01-1.18) | 0.033 | 1.01 (0.92-1.12) | 0.746 |
| **Alcohol drinking** | 1.07 (0.98-1.16) | 0.117 | 1.02 (0.92-1.13) | 0.693 |  | 1.10 (0.99-1.22) | 0.057 | 0.98 (0.87-1.10) | 0.704 |
| **Medical history** |  |  |  |  |  |  |  |  |  |
| CHD | 0.86 (0.81-0.91) | ＜0.001 | 0.89 (0.83-0.94) | ＜0.001 |  | 0.76 (0.71-0.81) | ＜0.001 | 0.83 (0.77-0.90) | ＜0.001 |
| Heart failure | 1.08 (0.99-1.17) | 0.092 | 1.08 (0.99-1.19) | 0.083 |  | 0.97 (0.87-1.07) | 0.482 | 0.99 (0.89-1.11) | 0.934 |
| Cerebrovascular disease | 0.86 (0.80-0.92) | ＜0.001 | 0.91 (0.85-0.98) | 0.016 |  | 0.78 (0.72-0.85) | ＜0.001 | 0.88 (0.80-0.96) | 0.003 |
| Diabetes mellitus | 0.80 (0.75-0.86) | ＜0.001 | 0.88 (0.82-0.94) | ＜0.001 |  | 0.72 (0.66-0.77) | ＜0.001 | 0.82 (0.76-0.89) | ＜0.001 |
| Previous bleeding | 0.84 (0.69-1.03) | 0.098 | 0.93 (0.75-1.14) | 0.474 |  | 0.70 (0.56-0.87) | 0.002 | 0.80 (0.64-1.01) | 0.062 |
| **Antihypertensive drugs** | 0.56 (0.53-0.59) | ＜0.001 | 0.60 (0.56-0.63) | ＜0.001 |  | 0.51 (0.48-0.55) | ＜0.001 | 0.56 (0.53-0.61) | ＜0.001 |
| **Etiological diagnosis of AF** |  |  |  |  |  |  |  |  |  |
| Valvular | 1.26 (1.15-1.38) | ＜0.001 | 1.16 (1.05-1.27) | 0.002 |  | 1.22 (1.09-1.36) | ＜0.001 | 1.11 (0.99-1.25) | 0.059 |
| Nonvalvular | 1 |  | 1 |  |  | 1 |  | 1 |  |
| **Types of AF** |  |  |  |  |  |  |  |  |  |
| Paroxysmal | 1 |  | 1 |  |  | 1 |  | 1 |  |
| First diagnosed | 1.32 (1.23-1.43) | ＜0.001 | 1.15 (1.07-1.24) | ＜0.001 |  | 1.24 (1.14-1.36) | ＜0.001 | 1.07 (0.98-1.18) | 0.129 |
| Persistent | 1.11 (1.04-1.19) | 0.002 | 1.12 (1.04-1.20) | 0.001 |  | 1.13 (1.04-1.22) | 0.004 | 1.17 (1.08-1.28) | ＜0.001 |
| Permanent | 1.26 (1.16-1.36) | ＜0.001 | 1.24 (1.14-1.35) | ＜0.001 |  | 1.27 (1.15-1.40) | ＜0.001 | 1.33 (1.20-1.48) | ＜0.001 |

Abbreviations: ACC/AHA, American College of Cardiology/American Heart Association; OR, odd ratio; CI, confidence interval; BMI, body mass index; CHD, coronary heart disease; AF, atrial fibrillation.

*BMI was available for 22 230 patients hospitalized with AF and hypertension based on the 2018 Chinese hypertension guideline; BMI was available for 26 693 hospitalized patients with AF and hypertension based on the 2017 ACC/AHA hypertension guideline.

Adjusted variables: age, sex, BMI, medical insurance, smoking, alcohol drinking, CHD, heart failure, cerebrovascular disease, diabetes mellitus, previous bleeding, antihypertensive drugs, etiological diagnosis of AF, and types of AF.

**Table 9. Investigators of the Improving Care for Cardiovascular Disease in China-Atrial Fibrillation (CCC-AF) project**

| **ID** | **Hospitals** | **Territories** | **Provinces** | **City** | **Investigator** |
| --- | --- | --- | --- | --- | --- |
| 1 | Peking University First Hospital | Northern China | Beijing | Beijing | Jie Jiang |
| 2 | Beijing Anzhen Hospital, Capital Medical University | Northern China | Beijing | Beijing | Shaoping Nie, Xiaohui Liu |
| 3 | The First Affiliated Hospital of Bengbu Medical College | Eastern China | Anhui | Bengbu | Honhju Wang |
| 4 | Beijing Friendship Hospital, Capital Medical University | Northern China | Beijing | Beijing | Hongwei Li |
| 5 | The First Affiliated Hospital of Chongqing Medical University | Southwest China | Chongqing | Chongqing | Suxin Luo |
| 6 | Changhai Hospital of Shanghai | Eastern China | Shanghai | Shanghai | Xianxian Zhao |
| 7 | Xinqiao Hospital, Third Military Medical University | Southwest China | Chongqing | Chongqing | Bin Cui, Lan Huang |
| 8 | Dongguan People's Hospital | Southern China | Guangdong | Dongguan | Jianfeng Ye |
| **ID** | **Hospitals** | **Territories** | **Provinces** | **City** | **Investigator** |
| 9 | Zhongda Hospital, Southeast University | Eastern China | Jiangsu | Nanjing | Genshan Ma |
| 10 | Gansu Provincial Hospital | Northwest China | Gansu | Lanzhou | Ping Xie |
| 11 | Guangdong General Hospital | Southern China | Guangdong | Guangzhou | Jiyan Chen |
| 12 | The First Affiliated Hospital of Guangxi Medical University | Southern China | Guangxi | Nanning | Lang Li |
| 13 | The People's Hospital of Guangxi Zhuang Autonomous Region | Southern China | Guangxi | Nanning | Yingzhong Lin |
| 14 | Panyu Hospital of Chinese Medicine | Southern China | Guangdong | Guangzhou | Jianhao Li |
| 15 | The Affiliated Hospital of Guizhou Medical University | Southwest China | Guizhou | Guiyang | Lirong Wu |
| 16 | The 2nd Affiliated Hosiptal of Harbin Medical University | Northeast China | Heilongjiang | Harbin | Bo Yu |
| 17 | Navy General Hospital | Northern China | Beijing | Beijing | Tianchang Li |
| **ID** | **Hospitals** | **Territories** | **Provinces** | **City** | **Investigator** |
| 18 | Haikou People's Hospital | Southern China | Hainan | Haikou | Moshui Chen |
| 19 | Hainan General Hospital | Southern China | Hainan | Haikou | Bin Li |
| 20 | The First Hospital of Handan | Northern China | Hebei | Handan | Shuanli Xin |
| 21 | Hebei General Hospital | Northern China | Hebei | Shijiazhuang | Xiaoyong Qi |
| 22 | The Second Hospital of Hebei Medical University | Northern China | Hebei | Shijiazhuang | Xianghua Fu |
| 23 | The First Affiliated Hospital of Henan University of Science and Technology | Central China | Henan | Luoyang | Pingshuan Dong |
| 24 | Henan Provincial People's Hospital | Central China | Henan | Zhengzhou | Chuanyu Gao |
| 25 | Chenzhou First People's Hospital | Central China | Hunan | Chenzhou | Qiaoqing Zhong |
| 26 | Hunan Provincial People's Hospital | Central China | Hunan | Changsha | Ying Guo |
| 27 | West China Hospital of Sichuan University | Northwest China | Sichuan | Chengdu | Xiaoping Chen |
| 28 | Huai'an First People's Hospital | Eastern China | Jiangsu | Huai'an | Shuren Ma |
| **ID** | **Hospitals** | **Territories** | **Provinces** | **City** | **Investigator** |
| 29 | The First Hospital of Jilin University | Northeast China | Jilin | Changchun | Yang Zheng |
| 30 | The Second Hospital of Jilin University | Northeast China | Jilin | Changchun | Bin Liu |
| 31 | Nanjing Drum Tower Hospital, The Affiliated Hospital of Nanjing University Medical School | Eastern China | Jiangsu | Nanjing | Biao Xu, Guangshu Han |
| 32 | Jiangsu Province Hospital | Eastern China | Jiangsu | Nanjing | Zhijian Yang |
| 33 | First Affiliated Hospital of the People's Liberation Army General Hospital | Northern China | Beijing | Beijing | Miao Tian |
| 34 | The First Affiliated Hospital of Lanzhou University | Northwest China | Gansu | Lanzhou | Zheng Zhang |
| 35 | The First Affiliated Hospital of Liaoning Medical University | Northeast China | Liaoning | Jinzhou | Guizhou Tao |
| 36 | China Meitan General Hospital | Northern China | Beijing | Beijing | Di Wu |
| 37 | The First Affiliated Hospital to Nanchang University | Eastern China | Jiangxi | Nanchang | Zeqi Zheng |
| **ID** | **Hospitals** | **Territories** | **Provinces** | **City** | **Investigator** |
| 38 | The Second Affiliated Hospital to Nanchang University | Eastern China | Jiangxi | Nanchang | Xiaoshu Cheng |
| 39 | Nanfang Hospital of Southern Medical University | Southern China | Guangdong | Guangzhou | Yuqing Hou |
| 40 | Inner Mongolia People's Hospital | Northern China | Inner Mongolia | Hohhot | Xingsheng Zhao |
| 41 | People’s Hospital of Qinghai Province | Northwest China | Qinghai | Xining | Rong Chang |
| 42 | Binzou City Center Hospital | Eastern China | Shandong | Binzhou | Lijun Meng |
| 43 | Shanxi Provincial People's Hospital | Northern China | Shanxi | Taiyuan | Chunlin Lai |
| 44 | Shanxi Cardiovascular Hospital | Northern China | Shanxi | Taiyuan | Bao Li |
| 45 | The Second Hospital of Shanxi Medical University | Northern China | Shanxi | Taiyuan | Zhiming Yang |
| 46 | The Ninth Hospital Affiliated to Shanghai Jiaotong University School of Medicine | Eastern China | Shanghai | Shanghai | Changqian Wang |
| 47 | Shanghai Sixth People's Hospital | Eastern China | Shanghai | Shanghai | Shixin Ma |
| **ID** | **Hospitals** | **Territories** | **Provinces** | **City** | **Investigator** |
| 48 | Tongren Hospital Affiliated to Shanghai Jiaotong University School of Medicine | Eastern China | Shanghai | Shanghai | Li Jiang |
| 49 | The General Hospital of Shenyang Military Region | Northeast China | Liaoning | Shenyang | Yaling Han |
| 50 | The Third Hospital of Shijiazhuang | Northern China | Hebei | Shijiazhuang | Zhenguo Ji |
| 51 | North Jiangsu People's Hospital | Eastern China | Jiangsu | Yangzhou | Shenghu He |
| 52 | General Hospital of TISCO | Northern China | Shanxi | Taiyuan | Huifeng Wang |
| 53 | Tianjin Chest Hospital | Northern China | Tianjin | Tianjin | Yin Liu |
| 54 | Teda International Cardiovascular Hospital | Northern China | Tianjin | Tianjin | Wenhua Lin |
| 55 | Tianjin Medical University General Hospital | Northern China | Tianjin | Tianjin | Yuemin Sun |
| 56 | Wuxi People's Hospital | Eastern China | Jiangsu | Wuxi | Zhenyu Yang |
| 57 | The First Affiliated Hospital of Xi’an Jiaotong University | Northwest China | Shaanxi | Xi'an | Zuyi Yuan |
| **ID** | **Hospitals** | **Territories** | **Provinces** | **City** | **Investigator** |
| 58 | Xijing Hospital | Northwest China | Shaanxi | Xi'an | Ling Tao |
| 59 | Southwest Hospital, Third Military Medical University | Southwest China | Chongqing | Chongqing | Zhiyuan Song |
| 60 | Hospital of Xinjiang Production & Construction Corps | Northwest China | Xinjiang | Urumchi | Junming Liu |
| 61 | The First Teaching Hospital of Xinjiang Medical University | Northwest China | Xinjiang | Urumchi | Yitong Ma |
| 62 | Xinjiang Uygur Autonomous Region People’s Hospital | Northwest China | Xinjiang | Urumchi | Guoqing Li |
| 63 | The Affiliated Hospital of Xuzhou Medical College | Eastern China | Jiangsu | Xuzhou | Zhirong Wang |
| 64 | People's Hospital of Yuxi City | Southwest China | Yunnan | Yuxi | Yinglu Hao |
| 65 | The Second People's Hospital of Yunnan Province | Southwest China | Yunnan | Kunming | Minghua Han |
| 66 | Sir Run Run Shaw Hospital, College of Medicine, Zhejiang University | Eastern China | Zhejiang | Hangzhou | Guosheng Fu |
| **ID** | **Hospitals** | **Territories** | **Provinces** | **City** | **Investigator** |
| 67 | The Second Affiliated Hospital of Zhengzhou University | Central China | Henan | Zhengzhou | Yulan Zhao |
| 68 | The First Affiliated Hospital of Zhengzhou University | Central China | Henan | Zhengzhou | Ling Li |
| 69 | The Third Xiangya Hospital of Central South University | Central China | Hunan | Changsha | Weihong Jiang |
| 70 | Sun Yat-sen Memorial Hospital, Sun Yat-sen University | Southern China | Guangdong | Guangzhou | Jingfeng Wang |
| 71 | Baogang Hospital | Northern China | Inner Mongolia | Baotou | Yongdong Li |
| 72 | Zhejiang Provincial Hospital of TCM | Eastern China | Zhejiang | Hangzhou | Wei Mao |
| 73 | Affiliated Hospital of Qinghai University | Northwest China | Qinghai | Xining | Weijun Liu |
| 74 | Anhui Provincial Hospital | Eastern China | Anhui | Hefei | Likun Ma |
| 75 | Anyang District Hospital | Central China | Henan | Anyang | Hui Liu |
| 76 | The Third the People‘s Hospital of Bengbu | Eastern China | Anhui | Bengbu | Gengsheng Sang |
| **ID** | **Hospitals** | **Territories** | **Provinces** | **City** | **Investigator** |
| 77 | Cangzhou Central Hospital | Northern China | Hebei | Cangzhou | Zesheng Xu |
| 78 | The First People's Hospital of Changde | Central China | Hunan | Changde | Yi Huang |
| 79 | Dalian Municipal Central Hospital | Northeast China | Liaoning | Dalian | Hailong Lin |
| 80 | The Second hospital of Dalian Medical University | Northeast China | Liaoning | Dalian | Peng Qu |
| 81 | The First Affiliated hospital of Dalian Medical University | Northeast China | Liaoning | Dalian | Yanzong Yang |
| 82 | Longyan First Hospital | Eastern China | Fujian | Longyan | Kaihong Chen |
| 83 | The First Affiliated Hospital of Fujian Medical University | Eastern China | Fujian | Fuzhou | Jinzi Su |
| 84 | Affiliated Hospital of Guangdong Medical College | Southern China | Guangdong | Guangzhou | Keng Wu |
| 85 | The First Affiliated Hospital of Guangzhou Medical College | Southern China | Guangdong | Guangzhou | Wei Wang |
| **ID** | **Hospitals** | **Territories** | **Provinces** | **City** | **Investigator** |
| 86 | The Third Affiliated Hospital of Guangzhou Medical College | Southern China | Guangdong | Guangzhou | Ximing Chen |
| 87 | Guizhou Provincial People's Hospital | Northwest China | Guizhou | Guiyang | Qiang Wu |
| 88 | The Central Hospital of Zhoukou | Central China | Henan | Zhoukou | Hualing Liu |
| 89 | The Central Hospital of Jilin | Northeast China | Jilin | Changchun | Shuangbin Li |
| 90 | The First People's Hospital of Jining | Eastern China | Shandong | Jining | Xiaofei Sun |
| 91 | Affiliated Hospital of Jiangsu University | Eastern China | Jiangsu | Zhenjiang | Jinchuan Yan |
| 92 | Jiangxi Provincial People's Hospital | Eastern China | Jiangxi | Nanchang | Lang Ji |
| 93 | The People's Hospital of Liaoning Province | Northeast China | Liaoning | Shenyang | Zhanquan Li |
| 94 | The First Affiliated Hospital of Liaoning University of Traditional Chinese Medicine | Northeast China | Liaoning | Shenyang | Ping Hou |
| 95 | Liaocheng People's Hospital | Eastern China | Shandong | Liaocheng | Chunyan Zhang |
| **ID** | **Hospitals** | **Territories** | **Provinces** | **City** | **Investigator** |
| 96 | Linyi People's Hospital | Eastern China | Shandong | Linyi | Zhihong Ou |
| 97 | Mudanjiang Cardiovascular Disease Hospital | Northeast China | Heilongjiang | Mudanjiang | Jianwen Liu |
| 98 | The First People's Hospital of Nanning City | Southern China | Guangxi | Nanning | Jinru Wei |
| 99 | Ningxia People's Hospital | Northwest China | Ningxia | Yinchuan | Hong Luan |
| 100 | Qingdao Municipal Hospital | Eastern China | Shandong | Qingdao | Jun Guan |
| 101 | Quanzhou First Hospital | Eastern China | Fujian | Quanzhou | Rong Lin |
| 102 | The First Affiliated Hospital of Xiamen University | Eastern China | Fujian | Xiamen | Qiang Xie |
| 103 | Xiamen Cardiovascular Disease Hospital | Eastern China | Fujian | Xiamen | Yan Wang |
| 104 | Qilu Hospital of Shandong University | Eastern China | Shandong | Jinan | Jifu Li |
| 105 | Yantaishan hospital | Eastern China | Shandong | Yantai | Juexin Fan |
| 106 | Zhongshan Hospital Affiliated to Fudan University | Eastern China | Shanghai | Shanghai | Junbo Ge |
| 107 | Shanghai East Hospital Affiliated to Tongji University | Eastern China | Shanghai | Shanghai | Xuebo Liu |
| **ID** | **Hospitals** | **Territories** | **Provinces** | **City** | **Investigator** |
| 108 | The Central Hospital of Shaoyang | Central China | Hunan | Shaoyang | Zewei Ouyang |
| 109 | Central Hospital Affiliated to Shenyang Medical College | Northeast China | Liaoning | Shenyang | Man Zhang, Kaiming Chen |
| 110 | The First Affiliated Hospital of Soochow University | Eastern China | Jiangsu | Suzhou | Xiangjun Yang |
| 111 | The Second Affiliated Hospital of Soochow University | Eastern China | Jiangsu | Suzhou | Weiting Xu |
| 112 | The Central Hospital of Taiyuan | Northern China | Shanxi | Taiyuan | Xiaoping Chen |
| 113 | Tangshan Gongren Hospital | Northern China | Hebei | Tangshan | Zheng Ji |
| 114 | The First Affiliated Hospital of Wannan Medical College | Eastern China | Anhui | Wuhu | Xingsheng Tang |
| 115 | The First Affiliated Hospital of Wenzhou Medical University | Eastern China | Zhejiang | Wenzhou | Weijian Huang |
| 116 | Wuzhou People's Hospital | Southern China | Guangxi | Wuzhou | Shaowu Ye |
| **ID** | **Hospitals** | **Territories** | **Provinces** | **City** | **Investigator** |
| 117 | Renmin Hospital of Wuhan University | Central China | Hubei | Wuhan | Hong Jiang |
| 118 | Xiangtan City Central Hospital | Central China | Hunan | Xiangtan | Lilong Tang |
| 119 | The Central Hospital of Xuzhou | Eastern China | Jiangsu | Xuzhou | Peiying Zhang |
| 120 | Affiliated Hospital of Yan'an University | Northwest China | Shaanxi | Yan'an | Xiaochuan Ma |
| 121 | Yancheng Third People's Hospital | Eastern China | Jiangsu | Yancheng | Chunyang Wu |
| 122 | Yichang Central Hospital | Central China | Hubei | Yichang | Jiawang Ding |
| 123 | The First People's Hospital of Yunnan Province (Kunhua Hospital) | Northwest China | Yunnan | Kunming | Hong Zhang |
| 124 | Hospital 463 of Chinese People's Liberation Army | Northeast China | Liaoning | Shenyang | Bosong Yang |
| 125 | The First Affiliated Hospital of China Medical University | Northeast China | Liaoning | Shenyang | Yingxian Sun |
| **ID** | **Hospitals** | **Territories** | **Provinces** | **City** | **Investigator** |
| 126 | The Fourth Affiliated Hospital of China Medical University | Northeast China | Liaoning | Shenyang | Yuanzhe Jin |
| 127 | The Second Xiangya Hospital of Central South University | Central China | Hunan | Changsha | Daoquan Peng |
| 128 | Xiangya Hospital Central South University | Central China | Hunan | Changsha | Tianlun Yang |
| 129 | Zhoushan People's Hospital | Eastern China | Zhejiang | Zhoushan | Guoxiong Chen |
| 130 | Chengdu Third People’s Hospital | Northwest China | Sichuan | Chengdu | Jiong Tang |
| 131 | Tangdu Hospital of The Fourth Military Medical University | Northwest China | Shaanxi | Xi'an | Xue Li |
| 132 | The First Hospital of Haerbin City | Northeast China | Heilongjiang | Harbin | Lin Wei |
| 133 | The First Affiliated Hospital of Jiamusi University | Northeast China | Heilongjiang | Jiamusi | Zhaofa He |
| 134 | The Central Hospital of Panzhihua | Northwest China | Sichuan | Panzhihua | Dawen Xu |
| **ID** | **Hospitals** | **Territories** | **Provinces** | **City** | **Investigator** |
| 135 | Wuhan Asia Heart Hospital | Central China | Hubei | Wuhan | Xi Su |
| 136 | Sichuan Provincial People’s Hospital | Northwest China | Sichuan | Chengdu | Jianhong Tao |
| 137 | The Central Hospital of Mianyang | Northwest China | Sichuan | Mianyang | Caidong Luo |
| 138 | The First Hospital of Jiamusi | Northeast China | Heilongjiang | Jiamusi | Guixia Zhang |
| 139 | Beijing Tsinghua Changgung Hospital | Northern China | Beijing | Beijing | Ping Zhang |
| 140 | Chongqing Hechuan District People’s Hospital | Southwest China | Chongqing | Chongqing | Xin Tang |
| 141 | Yuzhou City Central Hospital | Central China | Henan | Xuchang | Qinfeng Su |
| 142 | Jianshui County People’s Hospital | Southwest China | Yunnan | Honghe | Weiqing Fan |
| 143 | Dunhua City Hospital | Northeast China | Jilin | Yanbian | Fanju Meng |
| 144 | Shenyang City Electricity Central Hospital | Northeast China | Liaoning | Shenyang | Jing Xu |
| 145 | Shanghai Jingan District Shibei Hospital | Eastern China | Shanghai | Shanghai | Bin Wang |
| 146 | Beijing Fangshan District First Hospital | Northern China | Beijing | Beijing | Xuemei Peng |
| **ID** | **Hospitals** | **Territories** | **Provinces** | **City** | **Investigator** |
| 147 | Hebei Daming County People’s Hospital | Northern China | Hebei | Handan | Haiping Guo |
| 148 | Jiangsu Binhai County People’s Hospital | Eastern China | jiangsu | Yancheng | Yonglin Zhang |
| 149 | The First People’s Hospital of Longquanyi District | Southwest China | Sichuan | Chengdu | Wei Tuo |
| 150 | Guangxi Hengxian County People’s Hospital | Southern China | Guangxi | Nanning | Xianan Zhang |
| 151 | Hunan Changsha County First People’s Hospital | Central China | Hunan | Changsha | Siding Wang |
| 152 | People’s Hospital of Wugang | Central China | Hunan | Shaoyang | JiaoMei Yang |
| 153 | Longhui County People’s Hospital | Central China | Hunan | Shaoyang | Xiaojun Wang |
| 154 | Heilongjiang Fujin City Central Hospital | Northeast China | Heilongjiang | Jiamusi | Jiyan Yin |
| 155 | Dalian Fourth People’s Hospital | Northeast China | Liaoning | Dalian | Huifang Zhang |
| 156 | General Hospital of Guangzhou Military Command | Southern China | Guangdong | Guangzhou | Yanlie Zheng |
| 157 | The First People’s Hospital of Horqin District, Tongliao City | Northern China | Inner Mongolia | Tongliao | Junping Fang |
| **ID** | **Hospitals** | **Territories** | **Provinces** | **City** | **Investigator** |
| 158 | Guiyang Sixth People’s Hospital | Southwest China | Guizhou | Guiyang | Kalan Luo |
| 159 | Geological Mining Hospital of Hunan Province | Central China | Hunan | Changsha | Naiyi Liang |
| 160 | Zhangzhou Municipal Hospital of Fujian Province | Eastern China | Fujian | Zhangzhou | Changyong Liu |
| 161 | Jining City Yanzhou District People’s Hospital | Eastern China | Shandong | Jining | Jian Yang |
| 162 | The People’s Hospital Feixian | Eastern China | Shandong | Linyi | Honghua Deng |
| 163 | Tangshan City Fengrun District People’s Hospital | Northern China | Hebei | Tangshan | Lin Wang |
| 164 | Qian’an People’s Hospital | Northern China | Hebei | Tangshan | Yuheng Yang |
| 165 | Yuzhong County People’s Hospital | Northwest China | Gansu | Lanzhou | Xiaowei Peng |
| 166 | Baiyin Cite Center Hospital | Northwest China | Gansu | Baiyin | Fang Zhao |
| 167 | Mingguang People’s Hospital | Eastern China | Anhui | Chuzhou | Yong Li |
| 168 | Xihua County People’s Hospital | Central China | Henan | Zhoukou | Chuntong Wang |
| **ID** | **Hospitals** | **Territories** | **Provinces** | **City** | **Investigator** |
| 169 | Zhalantun People’s Hospital | Northern China | Inner Mongolia | Hulunbeier | Yuhua Zhu |
| 170 | Fengrun District Second People’s Hospital | Northern China | Hebei | Tangshan | Jingshan Zhao |
| 171 | Zhangping City Hospital | Eastern China | Fujian | Longyan | Jinxing Yi |
| 172 | The Eight Affiliated Hospital, Sun Yat-sen University | Southern China | Guangdong | Guangzhou | Nan Jia |
| 173 | The Second Affiliated Hospital of Qiqihar Medical Hospital | Northeast China | Heilongjiang | Qiqihar | Yanli Wang |
| 174 | Fuqing Cite Hospital | Eastern China | Fujian | Fuqing | Ping Chen |
| 175 | Wuhan University of Science and Technology Hospital | Central China | Hubei | Wuhan | Jing Hu |
| 176 | Baotou City Center Hospital | Northern China | Inner Mongolia | Baotou | Ruiping Zhao |
| 177 | Shanghai Jiading District Center Hospital | Eastern China | Shanghai | Shanghai | Xia Chen |
| **ID** | **Hospitals** | **Territories** | **Provinces** | **City** | **Investigator** |
| 178 | Shanghai Jiangwan Hospital | Eastern China | Shanghai | Shanghai | Aiping Li |
| 179 | Datong City Second People’s Hospital | Northern China | Shanxi | Datong | Xiaoqin Zhang |
| 180 | Shouyang County People’s Hospital | Northern China | Shanxi | Jinzhong | Buqiang Zhang |
| 181 | Xishan Coal Electricity Worker Ceneral Hospital | Northern China | Shanxi | Taiyuan | Suyan Zhou |
| 182 | Xixian People’s Hospital | Northern China | Shanxi | Linfen | Wenjun Yuan |
| 183 | Binyang People’s Hospital | Southern China | Guangxi | Nanning | Fudong Gan |
| 184 | Deqing People’s Hospital | Eastern China | Zhejiang | Huzhou | Fangfang Huang |
| 185 | Xinmi people’s hospital | Central China | Henan | Zhengzhou | Xiaolei Li |
| 186 | Dongguan Changping hospital | Southern China | Guangdong | Dongguan | Haiyun Lin |
| 187 | Gongyi people’s hospital | Central China | Henan | Zhengzhou | Tianmin Du |
| 188 | Ye County people’s hospital | Central China | Henan | Pingdingshan | Jie Yang |
| 189 | The second people’s hospital of Mengcheng | Eastern China | Anhui | Bozhou | Pengfei Zhang |
| **ID** | **Hospitals** | **Territories** | **Provinces** | **City** | **Investigator** |
| 190 | Nanpi People’s Hospital | Northern China | Hebei | Cangzhou | Hui Dong |
| 191 | Shimen People’s Hospital | Central China | Hunan | Changde | Chuanliang Liang |
| 192 | Tieli People’s Hospital | Northeast China | Heilongjiang | Yichun | Yanbo Niu |
| 193 | Sihui People’s Hospital | Southern China | Guangdong | Zhaoqing | Yuehua Huang |
| 194 | Chest Hospital of Xinjiang Uygur Autonomous Region | Northwest China | Xinjiang | Urumchi | Dongsheng Chai |
| 195 | Beian First People’s Hospital | Northeast China | Heilongjiang | Heihe | Dongyan Li |
| 196 | Zunhua People’s Hospital | Northern China | Hebei | Tangshan | Xiaoli Yang |
| 197 | Lujiang People’s Hospital | Eastern China | Anhui | Hefei | Qichun Wang |
| 198 | Qinyang People’s Hospital | Central China | Henan | Jiaozuo | Xiaowen Ma |
| 199 | Longmen People’s Hospital | Southern China | Guangdong | Huizhou | Yingchao Luo |
| 200 | Quyang Renji Hospital | Northern China | Hebei | Baoding | Congliang Zhang |
| 201 | Nenjiang People’s Hospital | Northeast China | Heilongjiang | Heihe | Shuhua Zhang |
| **ID** | **Hospitals** | **Territories** | **Provinces** | **City** | **Investigator** |
| 202 | Longjiang First People’s Hospital | Northeast China | Heilongjiang | Qiqihar | Yuhuan Shi |
| 203 | Li County Hospital of Traditional Chinese Medicine | Central China | Hunan | Changde | Songbai Li |
| 204 | Luan County People’s Hospital | Northern China | Hebei | Tangshan | Guo Li |
| 205 | Xinjin County Hospital of Traditional Chinese Medicine | Northwest China | Sichuan | Chengdu | Yingbi Su |
| 206 | Yulong Hospital | Southwest China | Yunnan | Lijiang | Zeyuan He |
| 207 | Yuncheng Hospital | Eastern China | Shandong | Heze | Jinglan Diao |
| 208 | Hepu People’s Hospital | Southern China | Guangxi | Beihai | Meisheng Lai |
| 209 | Duzishan Petrochemical Hospital | Northwest China | Xinjiang | Karamay | Shuqiu Qu |
| 210 | Guiding People’s Hospital | Southwest China | Guizhou | Qinan | Guoduo Chen |
| 211 | People’s Hospital of Rongchang District | Southwest China | Chongqing | Chongqing | Jie Chen |
| 212 | Ningbo First Hospital | Eastern China | Zhejiang | Ningbo | Huimin Chu |
| 213 | Ledong Second People’s Hospital | Southern China | Hainan | Ledong | Xiufeng Chen |
| **ID** | **Hospitals** | **Territories** | **Provinces** | **City** | **Investigator** |
| 214 | Guang’an People’s Hospital | Southwest China | Sichuan | Guang’an | Tian Tuo |
| 215 | Linfen People’s Hospital | Northern China | Shanxi | Linfen | Junping Deng |
| 216 | People’s Hospital of Bozhou District | Southwest China | Guizhou | Zunyi | Shengyong Chen |
| 217 | Dianjiang People’s Hospital | Southwest China | Chongqing | Chongqing | Yang Yu |
| 218 | First Affiliated Hospital of Harbin Medical University | Northeast China | Heilongjiang | Harbin | Yue Li |
| 219 | Haidong Ping’an District Hospital of Traditional Chinese Medicine | Northwest China | Qinghai | Haidong | Guoqin Xin |
| 220 | Ningjin People’s Hospital | Eastern China | Shandong | Dezhou | Tao Zhang |
| 221 | Yutian Hospital | Northern China | Hebei | Tangshan | Xiaoyun Feng |
| 222 | Yanting People’s Hospital | Southwest China | Sichuan | Mianyang | Mingcheng Bai |
| 223 | The Fourth Affiliated Hospital Zhejiang University School of Medicine | Eastern China | Zhejiang | Yiwu | Shudong Xia |
| 224 | Zhongda Hospital, Southeast University (Jiangbei) | Eastern China | Jiangsu | Nanjing | Jiayi Tong |
| **ID** | **Hospitals** | **Territories** | **Provinces** | **City** | **Investigator** |
| 225 | Wuxi Xishan People’s Hospital | Eastern China | Jiangsu | Wuxi | Xudong Li |
| 226 | Dongfeng Hospital | Northeast China | Jilin | Liaoyuan | Wei Liu |
| 227 | Zhijin People’s Hospital | Southwest China | Guizhou | Bijie | Zhongshan Wang |
| 228 | Huaiyang People’s Hospital | Central China | Henan | Zhoukou | Li Wei |
| 229 | Suizhou Central Hospital | Central China | Hubei | Suizhou | Fengwei Li |
| 230 | Tonglu First People’s Hospital | Eastern China | Zhejiang | Hangzhou | Xiaolan Li |
| 231 | Xiantao First People’s Hospital | Central China | Hubei | Xiantao | Dongmei Zhu |
| 232 | Honghu People’s Hospital | Central China | Hubei | Jingzhou | Hong Liu |
| 233 | Affilioted Hospital of North Sichuan Medical College | Northwest China | Sichuan | Nanchong | Zhan Lv |
| 234 | Guangyuan Central Hospital | Northwest China | Sichuan | Guangyuan | Bing Fu |
| 235 | Dazhou Central Hospital | Northwest China | Sichuan | Dazhou | Yong Guo |
| 236 | Nanchong Central Hospital | Northwest China | Sichuan | Nanchong | Tao Liu |


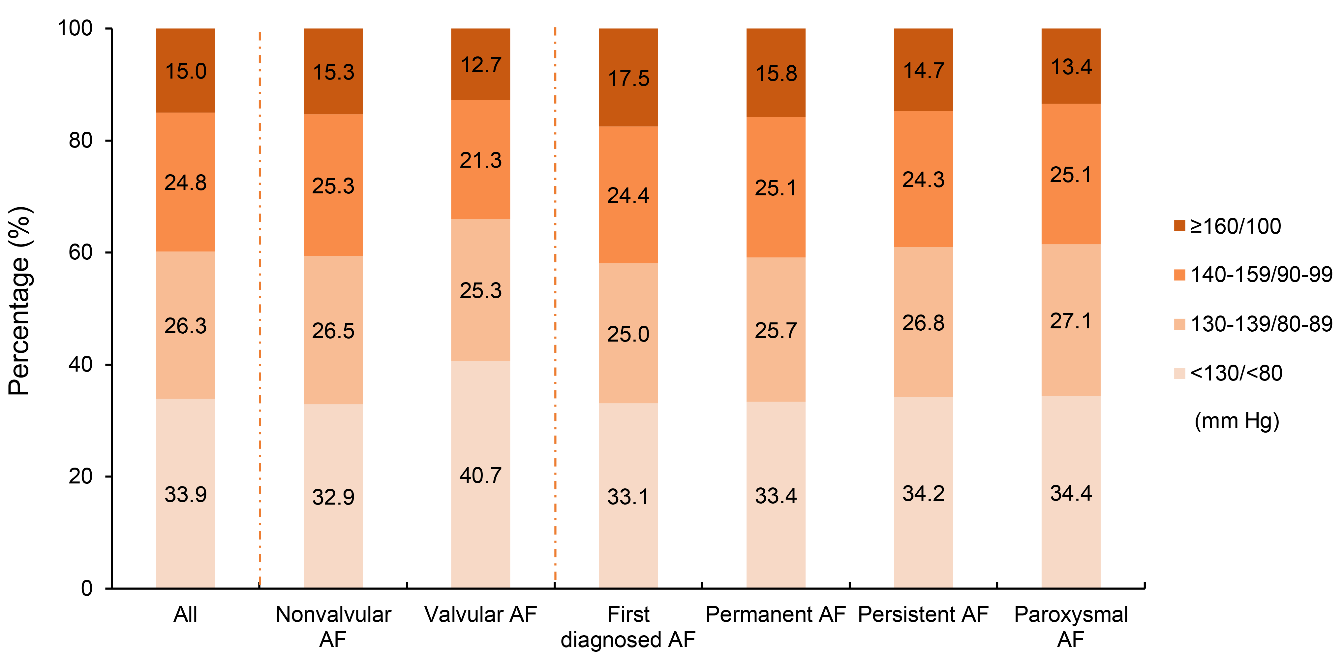


**Figure 1.** Proportions of patients hospitalized with atrial fibrillation (AF) with different blood pressure levels in China.


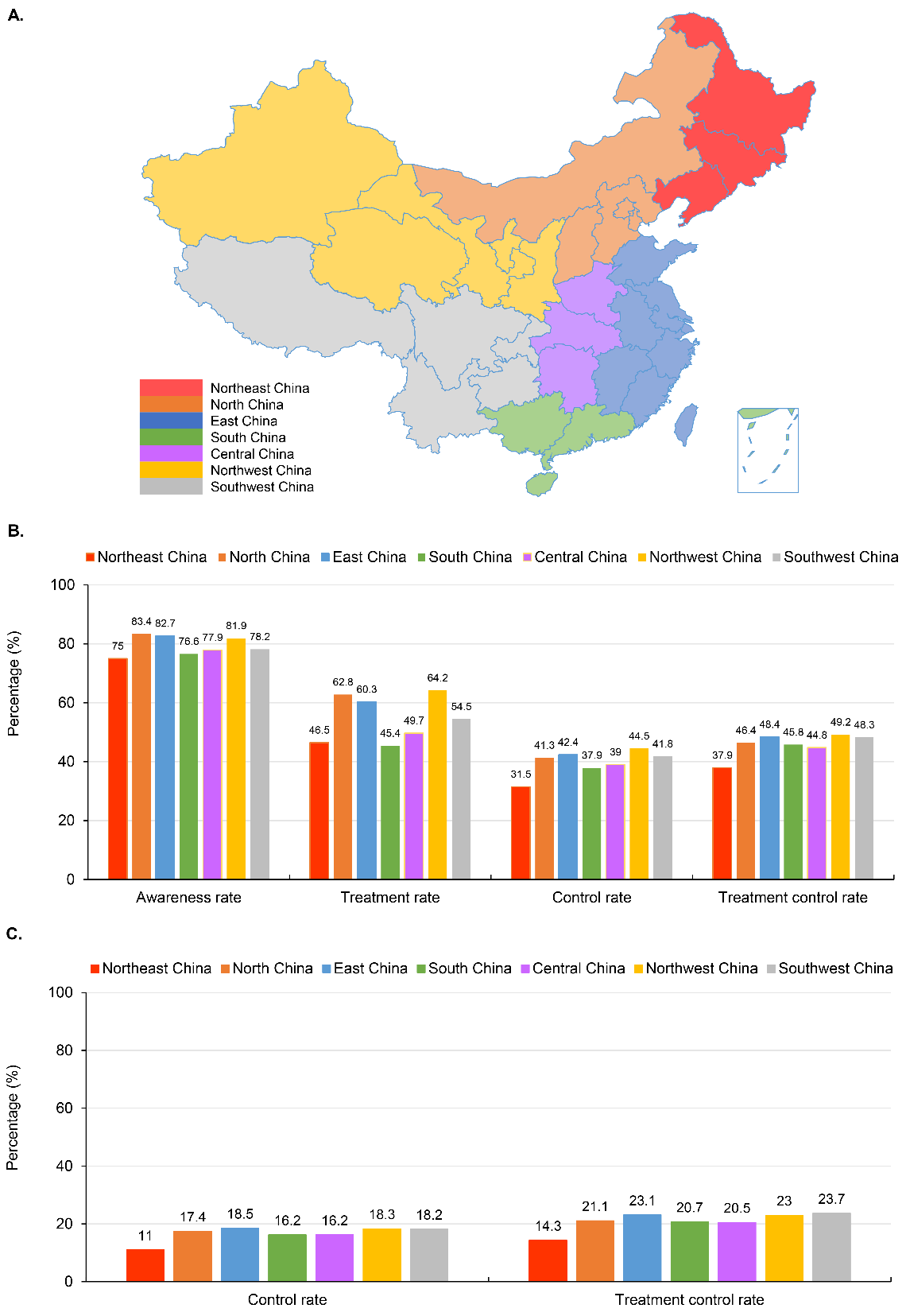


**Figure 2.** **Awareness, treatment, and control rates of hypertension in patients hospitalized with atrial fibrillation (AF) in different regions of China.**

(A) distribution of the seven regions in China; (B) according to the 2018 Chinese hypertension guidelines; (C) according to the 2017 American College of Cardiology/American Heart Association (ACC/AHA) hypertension guidelines.
